# Optimal Deployment of Gonorrhoea Point-of-Care Tests: Modeling the Potential Impact of Diagnostic Confirmation Testing and Screening Strategies Across Five Priority Populations in Kenya

**DOI:** 10.1093/infdis/jiag162

**Published:** 2026-03-17

**Authors:** Julia Michalow, Anne Cori, Joshua Kimani, Parinita Bhattacharjee, Marie-Claude Boily, Jeffrey W Imai-Eaton

**Affiliations:** MRC Centre for Global Infectious Disease Analysis, School of Public Health, Imperial College London, London, United Kingdom; MRC Centre for Global Infectious Disease Analysis, School of Public Health, Imperial College London, London, United Kingdom; Partners for Health and Development in Africa (PHDA), Nairobi, Kenya; Partners for Health and Development in Africa (PHDA), Nairobi, Kenya; Institute for Global Public Health, University of Manitoba, Winnipeg, Manitoba, Canada; MRC Centre for Global Infectious Disease Analysis, School of Public Health, Imperial College London, London, United Kingdom; MRC Centre for Global Infectious Disease Analysis, School of Public Health, Imperial College London, London, United Kingdom; Center for Communicable Disease Dynamics, Department of Epidemiology, Harvard T.H. Chan School of Public Health, Boston, Massachusetts, USA

**Keywords:** gonorrhea, point-of-care testing, syndromic management, diagnostic confirmation, screening strategies

## Abstract

**Background:**

Gonorrhea treatment in sub-Saharan Africa relies on syndromic management, which has poor diagnostic performance and misses asymptomatic infections. Point-of-care tests (POCTs) could address these limitations, but anticipated supply constraints necessitate strategic allocation to maximize impact.

**Methods:**

We developed a deterministic compartmental model of gonorrhea transmission in Kenya to evaluate allocating POCTs for diagnostic confirmation of symptomatic care attendees versus screening of routine healthcare service attendees across five priority populations: female sex workers (FSW), their male clients (CFSW), pregnant women, adolescent girls and young women, or total population men. We modeled constrained and unrestricted POCT availability during 2025–2030, and estimated infections averted relative to baseline syndromic management. Quality-adjusted life years (QALYs) gained were quantified using probability tree models.

**Results:**

At baseline, incidence was highest among FSW (11.9 [UI: 5.7–18.6] per 100 per year) and CFSW (13.1 [6.9–24.8]), while most QALY losses (80.6% [76.1%–83.8%]) were among pregnant women and their infants. With constrained POCTs (sufficient to test 0.1% of adults annually), diagnostic confirmation averted the most transmission when among symptomatic FSW (2.1% [0.6%–5.6%] of infections) or CFSW (2.2% [0.8%–5.3%]), and the most morbidity when among symptomatic pregnant women (3.5% [1.8%–7.2%] of QALY losses). Screening averted <1% of infections or QALY losses across populations. With unrestricted POCTs, screening had larger absolute impacts but lower per-test efficiency than diagnostic confirmation.

**Conclusions:**

Our modeling supports prioritizing diagnostic confirmation over screening, consistent with WHO guidance to strengthen etiologic diagnosis within syndromic care. Diagnostic testing among symptomatic pregnant women had the largest impact on mitigating gonorrhea-related morbidity.

## Teaser

Gonorrhea point-of-care testing maximized health impact when used for diagnostic confirmation within syndromic management rather than asymptomatic screening, based on modeling in Kenya. Testing symptomatic pregnant women averted the most morbidity, whereas testing higher-incidence populations averted the most transmission.

The World Health Organization (WHO) classifies *Neisseria gonorrhoeae* as a high-priority pathogen due to its global burden and rapid emergence of antimicrobial resistance (AMR) [1]. Gonorrhea disproportionately affects women, causing reproductive-tract complications such as pelvic inflammatory disease and infertility, and, when acquired during pregnancy, stillbirth and adverse neonatal outcomes [2, 3]. Prevalence in sub-Saharan Africa (SSA) is more than double the global average [4, 5].

Syndromic management, involving presumptive treatment based on symptoms, remains the primary approach for treating symptomatic sexually transmitted infections (STIs) in SSA. The approach has poor sensitivity and specificity for cervical infections including gonorrhea, resulting in missed infections and unnecessary treatment [6]. World Health Organization therefore recommends integrating molecular diagnostics into syndromic algorithms [6] and recently recommended targeted gonorrhea screening of priority populations in high-prevalence settings, given most infections are asymptomatic [7]. However, neither strategy has been implemented at scale in SSA, largely due to health system and financial constraints [8].

Point-of-care tests (POCTs) could expand access to diagnostic testing and screening. Existing gonorrhea POCTs fall short of WHO target product profile (TPP) criteria for cost, turnaround time, and accessibility, but new platforms are in development [9, 10]. Supply and resource constraints will likely necessitate strategic allocation of new POCTs [9, 11]. Key tradeoffs include: prioritizing incidence reduction versus morbidity reduction; prioritizing diagnostic confirmation among symptomatic individuals versus screening asymptomatic individuals; and within these, prioritizing men, who are more likely to experience symptoms [12], versus women, who are often inadequately managed under syndromic care and face greater risks of reproductive and pregnancy-related sequelae [6]. Previous modeling in SSA has evaluated diagnostic testing [13, 14] or screening [15, 16], but has not directly compared these strategies or considered constrained POCT availability.

To address these gaps, we modeled strategies for allocating gonorrhea POCTs to maximize reductions in incidence or morbidity in Kenya from 2025 to 2030. We evaluated diagnostic versus screening strategies across five priority populations [17, 18]: female sex workers (FSW), their male clients (CFSW), pregnant women, sexually active adolescent girls and young women (AGYW), and men in the total population. We focused on heterosexual transmission dynamics to examine the tradeoff between transmission control and morbidity reduction, as population health burden is concentrated among women in this setting [2].

## METHODS

### Compartmental Model Structure

We developed a deterministic compartmental model of heterosexual gonorrhea transmission among 15–49-year-olds in Kenya (Supplementary Text 1.1.1, Supplementary Figure 1). The model represented an open population stratified by sex (male, female), sexual risk (low, intermediate, high [FSW or CFSW]), age (15–24 years, 25–49 years), and gestational status (nonpregnant, pregnant). Individuals entered the model at 15–24 years as not yet sexually active, transitioning to sexual activity upon debut or aging to 25–49 years. Females transitioned between gestational states based on fertility rates. Exit was through all-cause mortality or ageing out at 50 years. Individuals did not transition between sexual risk groups.

Sexual partner acquisition rates were heterogeneous. Groups mixed across all strata proportionately to these rates, except FSW partnered exclusively with CFSW, while CFSW partnered with any female risk group (Supplementary Text 1.1.2). The force of infection incorporated mixing patterns, coital frequency, condom use, per-act transmission probability, and infection prevalence among partners (Supplementary Text 1.1.3). After infection, individuals were either asymptomatic or symptomatic. Symptomatic individuals had a probability of accessing care. Infections resolved rapidly with treatment or more slowly through natural clearance. We assumed no immunity and did not distinguish anatomical sites of infection.

We modeled three diagnostic and treatment scenarios (Supplementary Text 1.1.4, Supplementary Figure 1), assuming ceftriaxone-based dual therapy [19] was fully effective and available following diagnosis. AMR and treatment failure were not incorporated. In the baseline scenario, syndromic management was provided to individuals accessing care for vaginal or urethral discharge. A proportion were treated according to the algorithm's sensitivity. Symptomatic individuals who did not access care and asymptomatic individuals were not treated. In the diagnostic confirmation scenario, POCTs replaced syndromic management for a proportion of symptomatic care attendees. Those tested were diagnosed and treated according to test sensitivity, while those not tested were managed syndromically. Tests were distributed across all symptomatic cases regardless of etiology, with annual eligibility estimated using the gonorrhea etiologic proportion (proportion of discharge cases caused by gonorrhea). In the screening scenario, POCTs were offered to routine primary healthcare or antenatal care attendees, with tests allocated according to population-specific attendance probabilities. Screen-positive individuals were treated. Syndromic management continued for symptomatic care attendees.

### Model Parametrization and Calibration

The model incorporated 23 parameter types, which could vary by sex, risk, age, and/or gestational status. Nine were fixed and fourteen groups were calibrated, yielding 41 total calibrated parameters. Calibrated parameters had uniform prior distributions (Supplementary Text 1.2). Demographic parameters were from UN World Population Prospects 2024 (Supplementary Text 1.2.1, Supplementary Table 1) [20]. Sexual behavior, mixing, and care-seeking parameters for the general population were from Kenya Demographic and Health Surveys [21–24] and the Kenya Population-based HIV Impact Assessment [25], and from published studies for FSW and CFSW (Supplementary Text 1.2.2, Supplementary Table 2). Natural history parameters were from literature (Supplementary Table 2).

The model baseline scenario was calibrated to four outcomes: (i) gonorrhea prevalence among sexually active females (target range: 1.0%–3.5%), (ii) gonorrhea prevalence among FSW (2.0%–6.5%), (iii) male-to-female gonorrhea prevalence ratio (0.60–1.00), and (iv) male-to-female ratio for all-cause symptomatic case rates (0.35–0.60; Supplementary Text 1.3.1, Supplementary Table 3). Target ranges for prevalence outcomes came from a meta-regression of studies in Kenya between 2000 and 2025 (Supplementary Text 1.3.2, Supplementary Figure 2, Supplementary Table 4). The third was from a systematic review and meta-regression in SSA [5], and the fourth from DHS [21–24]. We generated 50,000 candidate parameter combinations from prior distributions (Supplementary Table 2) using Latin Hypercube Sampling. Parameter combinations were retained if equilibrium outputs fell within all calibration ranges.

### Morbidity and Health Outcomes

We quantified health losses from acute infection and sequelae accruing over the lifetime using post hoc quality-adjusted life years (QALY). In men, all symptomatic infections caused urethritis, and untreated infection could cause epididymo-orchitis [26]. In nonpregnant women, pelvic inflammatory disease (PID) could follow any infection and progress to combinations of chronic pelvic pain (CPP), ectopic pregnancy (EP), and tubal factor infertility (TFI) [26]. In pregnant women, untreated infection could cause stillbirth, low birth weight (LBW), neonatal pneumonia (NP), or ophthalmia neonatorum (ON) [2, 3], but not PID or its sequelae.

Quality-adjusted life years losses following infection were calculated as the probability of each outcome multiplied by its duration and utility decrement following assumptions from Li et al. [26] for men and nonpregnant women and a newly developed framework for pregnant women (Supplementary Text 1.4.1, Supplementary Figure 3). Acute infections (urethritis, cervicitis) were quantified as short-term decrements during an infection episode, while sequelae were quantified as longer-term losses per incident infection. PID risk increased with infection duration at an annual progression rate [26]. Co-occurring PID sequelae (CPP, EP, TFI) were jointly modeled to capture overlapping durations and combined utility losses (Supplementary Text 1.4.2) [26].

Probabilities, durations, and utility weights were from published literature (Supplementary Table 5). We estimated stillbirth and LBW probabilities among untreated pregnant women by applying published odds ratios to background population prevalence estimates (Supplementary Text 1.4.3, Supplementary Table 6) [3, 27, 28]. Sequelae durations varied by outcome (Supplementary Table 5). CPP and TFI were chronic sequelae manifesting 5 years post-infection and persisting for 10 years [26, 29]. Stillbirth-associated losses extended over life expectancy (utility weight of 0), and were thus valued equivalent to neonatal deaths [30]. Mothers of stillborn infants or infants with adverse outcomes were assigned utility decrements for psychological impacts (Supplementary Table 5) [31, 32].

For infections acquired each year, we summed all QALY losses occurring over the infected individual's remaining lifetime. Lifetime losses were discounted to the year of infection acquisition at 3% annually (Supplementary Text 1.4.4, Supplementary Table 7).

### Intervention Scenarios

We modeled introduction of POCT for diagnostic confirmation or screening among five priority populations (FSW, CFSW, pregnant women, sexually active nonpregnant AGYW, and total population men). Strategies were modeled independently per priority population, yielding 10 scenarios, though population groups were not mutually exclusive. To assess impact under constrained availability, we assumed 25,000 POCTs annually (approximately 0.1% of adults). To assess maximum potential impact, we also modeled hypothetical unrestricted POCT supply. POCTs were assumed to meet WHO optimal TPP (95% sensitivity, 98% specificity) [33]. We introduced interventions in 2025 and maintained them through 2030.

We compared intervention outcomes to baseline for all posterior parameter combinations. Outcomes over the 6-year intervention period were POCT coverage among the eligible population, effectiveness (proportion of infections averted or proportion of QALYs gained), and efficiency (infections averted or QALYs gained per test used). Results are reported as medians with uncertainty intervals (UIs; 2.5th–97.5th percentiles).

### Sensitivity Analyses

To identify parameters influencing population prioritization, we ranked priority populations by proportion of infections averted under each strategy, and calculated partial rank correlation coefficients (PRCCs) between posterior parameter values and these ranks [34]. To assess sensitivity of results to pregnancy-related sequelae assumptions, we (i) reduced the lifetime QALY loss duration to 50% of life expectancy for stillbirths and to 10 years for LBW, and (ii) excluded stillbirth-associated losses entirely. Sensitivity analyses were for the constrained POCT availability scenario.

Analyses were conducted in R v4.4.1. Ordinary differential equations were implemented with *odin* (v1.5.10) [35], Latin Hypercube Sampling with *lhs* (v1.2.0) [36], and prevalence meta-regression for calibration outcomes with *glmmTMB* (v1.1.9) [37].

## RESULTS

### Model Calibration

Of 50 000 sampled parameter combinations, 536 were retained after calibration (Supplementary Text 2.1, Supplementary Figures 4-7). Baseline gonorrhea prevalence among all sexually active 15–49-year-olds was 1.8% (UI: 1.1%–3.3%; target: 1.0%–3.5%) for females and 1.3% (UI:0.7%–2.3%) for males. Prevalence was highest among FSW (4.7% [UI: 2.4%–6.5%], target: 2.0%–6.5%) and CFSW (3.6% [2.0%–6.7%]), followed by pregnant women (1.9% [1.1%–3.6%]) and AGYW (1.8% [1.1%–3.3%]; Supplementary Table 8).

### Gonorrhea Burden Before Intervention

**Figure 1.**
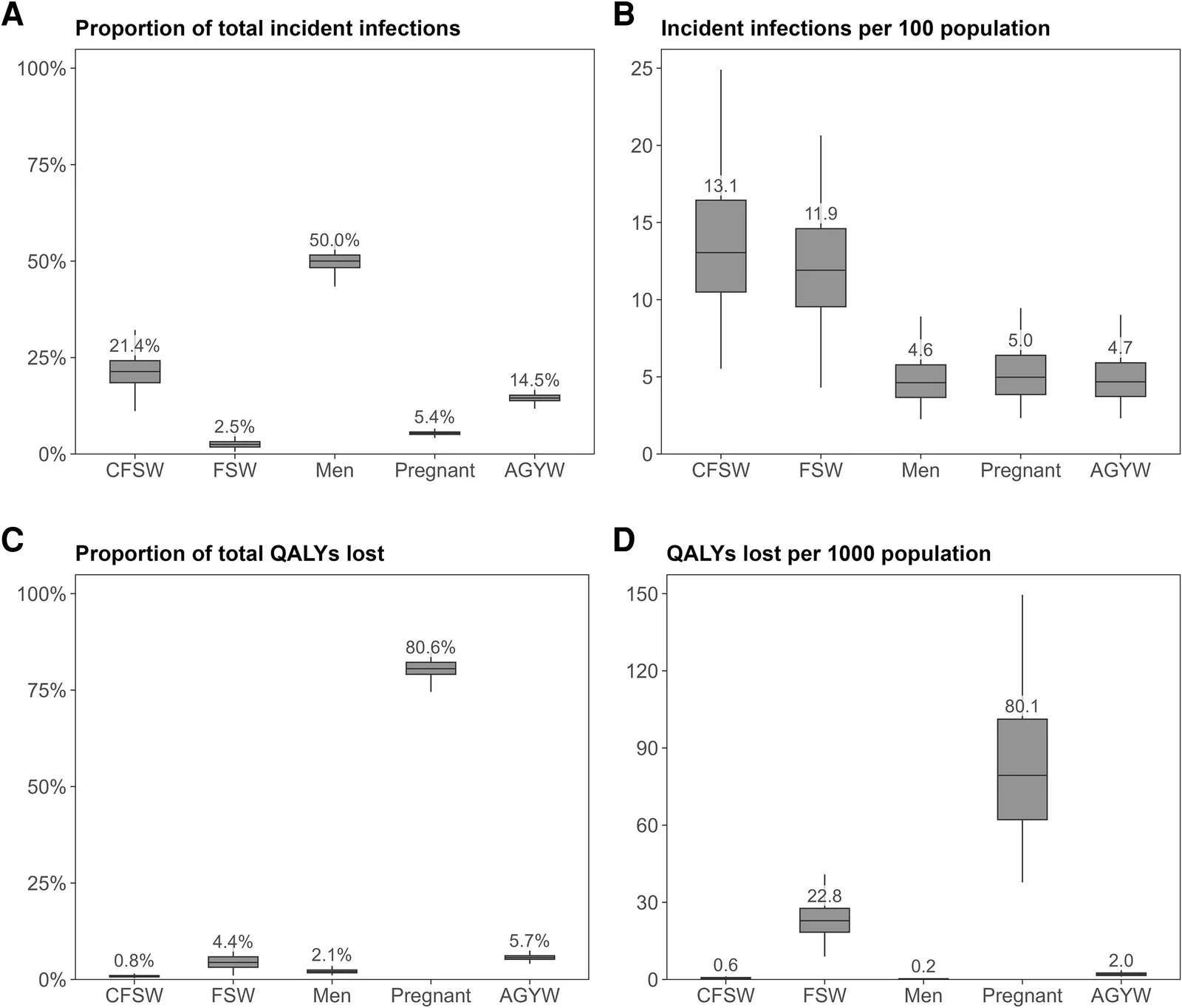
Gonorrhoea burden before intervention, 2025. Outcomes per priority population prior to intervention in 2025: (*A*) proportion of total incident infections, (*B*) incident infections per 100 individuals, (*C*) proportion of total lifetime QALYs lost, (*D*) lifetime QALYs lost per 1000 individuals. Priority populations are not mutually exclusive; proportions in panels A and C therefore do not sum to 100%. Boxplots show median (horizontal line), interquartile range (box), and uncertainty interval (whiskers). Abbreviations: AGYW, sexually active nonpregnant adolescent girls and young women; CFSW, clients of female sex workers; FSW, female sex workers; QALY, quality-adjusted life year.

We estimated 1.1 million (UI: 0.6–2.9 million) incident infections in 2025, 50.0% (45.4%–55.0%) of which were among men (Figure 1*A*). CFSW and FSW were 7.4% (5.3%–10.1%) and 1.0% (0.5%–1.5%) of the total population (both sexes), respectively, and contributed 21.4% (13.7%–29.3%) and 2.5% (1.0%–5.6%) of total incident infections. Gonorrhea incidence rate was highest among CFSW (13.1 [6.9–24.8] per 100 individuals) and FSW (11.9 [5.7–18.6]), and similar for pregnant women (5.0 [2.8–9.1]), AGYW (4.7 [2.8–8.5]), and total population men (4.6 [2.7–8.3]; Figure 1*B*). 91.7% [87.9%–94.9%] of infections were untreated among females compared with 63.7% [50.6%–73.8%] among males (Supplementary Figure 8).

We estimated 115 105 (66 430–207 620) discounted lifetime QALYs lost due to infections acquired in 2025, of which 80.6% (76.1%–83.8%) were among pregnant women and their infants (Figure 1*C*), mainly from stillbirth (70.0%) and LBW among infants (26.5%; Supplementary Figure 9). QALYs lost per 1000 individuals were highest among pregnant women (80.1 [UI: 45.1–145.0]), followed by FSW (22.8 [11.1–36.0]), AGYW (2.0 [1.2–3.7]), CFSW (0.6 [0.3–1.2]), and men (0.2 [0.1–0.5]; Figure 1*C*).

### Impact of Constrained Point-of-Care Tests

With 25 000 POCTs available annually between 2025 and 2030, the proportion of individuals with discharge symptoms who received diagnostic confirmation testing ranged from 8.0% (UI: 3.9%–16.2%) among symptomatic total population men to 66.8% (26.0%–100.0%) among FSW. The proportion of routine healthcare attendees who received screening ranged from 0.14% (0.14%–0.15%) among men to 5.8% (3.9%–11.1%) among FSW (Supplementary Table 9).

**Figure 2.**
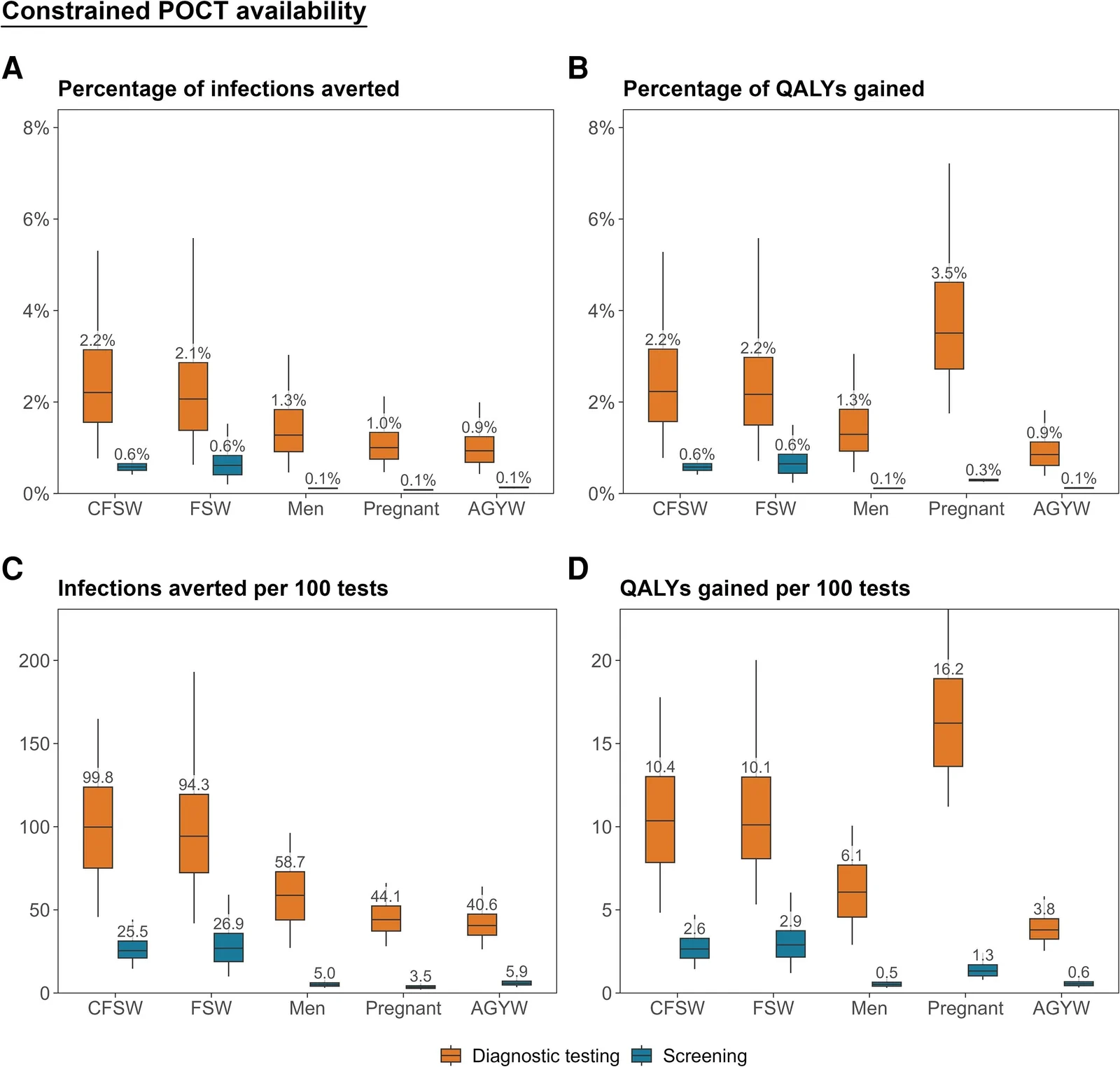
Population-level impact of diagnostic confirmation and screening strategies among each priority population under constrained POCT availability, 2025–2030. Population-level impact of deploying a constrained supply of gonorrhea POCTs among each of five priority populations between 2025 and 2030 through either diagnostic confirmation testing of individuals accessing care for genital discharge, or screening of attendees at routine healthcare services. Impact measured as (*A*) percentage of incident infections averted, (*B*) percentage of QALYs gained, (*C*) incident infections averted per 100 POCTs used, and (*D*) QALYs gained per 100 POCTs used, relative to the baseline with syndromic management only. Boxplots represent the median (horizontal line), interquartile range (box), and uncertainty interval (whiskers). Abbreviations: AGYW, sexually active nonpregnant adolescent girls and young women; CFSW, clients of female sex workers; FSW, female sex workers; POCT, point-of-care test; QALY, quality-adjusted life year.

Diagnostic confirmation was estimated to avert 2.2% (UI: 0.8%–5.3%) of all incident infections between 2025 and 2030 when modeled among CFSW, 2.1% (0.6%–5.6%) when among FSW, 1.3% (0.5%–3.0%) when among men, 1.0% (0.5%–2.1%) when among pregnant women, and 0.9% (0.4%–2.0%) when among AGYW (Figure 2*A*, Supplementary Table 9). The impact of screening was smaller, averting 0.6% (0.2%–1.5%) of all incident infections when modeled among FSW and 0.6% (0.4%–0.8%) among CFSW, followed by 0.13% (0.11%–0.16%) among AGYW, 0.12% (0.10%–0.13%) among men, and 0.08% (0.06%–0.10%) among pregnant women (Figure 2*A*).

In contrast, estimated QALY gains were largest for diagnostic confirmation among pregnant women (Figure 2*B*, Supplementary Table 9), averting 3.5% (1.8%–7.2%) of population-wide lifetime QALY losses, reflecting the high burden of pregnancy and neonatal sequelae. This exceeded the impact of testing FSW (2.2% [0.7%–5.6%]) or CFSW (2.2% [0.8%–5.3%]), despite those strategies averting twice as many incident infections. Screening pregnant women averted an estimated 0.29% (0.25%–0.34%) of QALY losses, intermediate between screening high- and lower-risk groups.

Patterns were consistent whether assessed by total impact (Figure 2*A* and 2*B*), or infections and QALY losses averted per test (Figure 2*C* and 2*D*).

### Impact of Unrestricted Point-of-Care Tests

**Figure 3.**
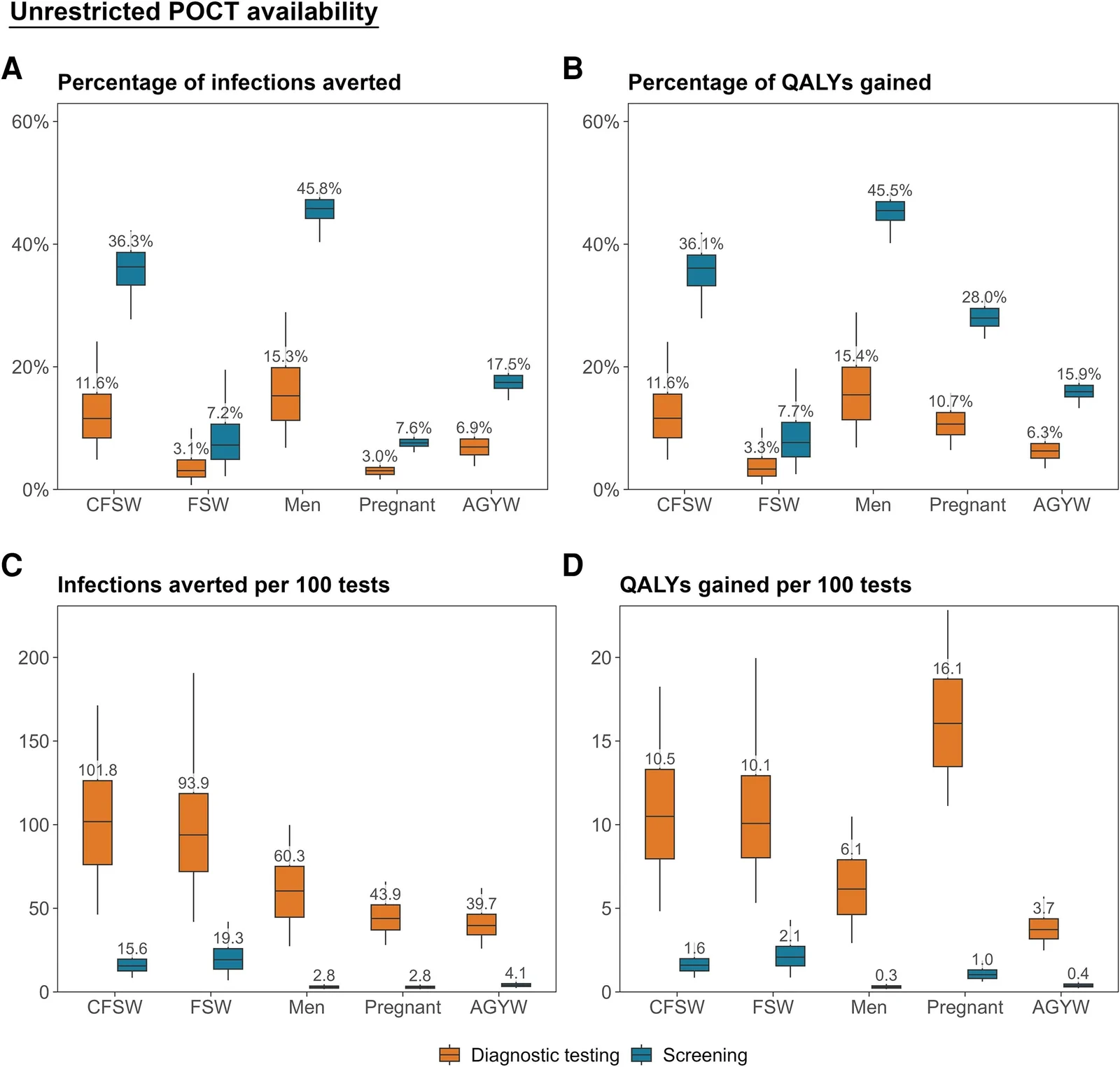
Population-level impact of diagnostic confirmation and screening strategies among each priority population under unrestricted POCT availability, 2025–2030. Population-level impact of deploying an unlimited supply of gonorrhoea POCTs among each of five priority populations between 2025 and 2030 through either diagnostic confirmation testing of individuals accessing care for genital discharge, or screening of attendees at routine healthcare services. Impact measured as (*A*) percentage of incident infections averted, (*B*) percentage of QALYs gained, (*C*) incident infections averted per 100 POCTs used and (*D*) QALYs gained per 100 POCTs used, relative to the baseline with syndromic management only. Boxplots represent the median (horizontal line), interquartile range (box), and uncertainty interval (whiskers). Abbreviations: AGYW, sexually active nonpregnant adolescent girls and young women; CFSW, clients of female sex workers; FSW, female sex workers; POCT, point-of-care test; QALY, quality-adjusted life year.

With unrestricted POCTs between 2025 and 2030, diagnostic confirmation averted 15.3% (UI: 6.8%–28.9%) of all incident infections when modeled among total population men, followed by 11.6% (4.9%–24.1%) among CFSW, 6.9% (3.8%–10.7%) among AGYW, 3.1% (0.7%–10.0%) among FSW, and 3.0% (1.6%–5.0%) among pregnant women (Figure 3*A*, Supplementary Table 10). The relative pattern of QALYs gained across populations was similar, except diagnostic confirmation among pregnant women averted 10.7% (6.4%–15.8%) of total lifetime QALY losses (Figure 3*B*).

Screening was estimated to avert more infections and QALY losses than diagnostic confirmation across all populations (Figure 3*A* and 3*B*; Supplementary Table 10) but was consistently less efficient in terms of infections averted per test (Figure 3*C* and 3*D*). Screening males had the largest modeled impact, averting 45.8% (40.3%–49.1%) of infections and 45.5% (40.1%–48.5%) of QALY losses when conducted among total population men, and 36.3% (27.7%–42.2%) and 36.1% (27.9%–41.9%) when among CFSW, respectively. However, efficiency was far lower for men (2.8 [1.7–4.9] infections averted and 0.3 [0.2–0.5] QALYs gained per 100 tests) than CFSW (15.6 [8.5–28.6] and 1.6 [0.9–3.0]). Screening pregnant women averted 28.0% (24.6%–31.8%) of estimated QALY losses but with lower efficiency than higher-risk groups.

Diagnostic confirmation efficiency was similar under constrained and unrestricted POCT availability across all populations, whereas screening efficiency was consistently higher under constrained availability (Figures 2*C* and 2*D* and 3*C* and 3*D*).

### Sensitivity Analyses

When prioritizing populations for diagnostic confirmation based on total infections averted, only 24% (n = 131/536) of parameter combinations ranked populations in the same order of impact as median estimates. However, in 89% of parameter combinations either CFSW (n = 299/536, 55.8%) or FSW (n = 234/536, 43.7%) were the highest-impact population for testing. Prioritization was strongly associated with the sensitivity of urethral discharge management: higher sensitivity reduced the marginal benefit of POCTs for male populations (PRCC: 0.75 [UI: 0.72–0.79] total population men; 0.67 [0.62–0.72] CFSW) and increased the relative prioritization of female populations (Supplementary Figure 10A). For screening, 53% (n = 282/536) of parameter combinations yielded the same population ranking as median estimates, with either FSW (n = 297/536, 55.4%) or CFSW (n = 239/536, 44.6%) ranked as highest impact. Higher relative numbers of sexual partnerships among FSW increased FSW prioritization (−0.69 [−0.75; −0.66]), while decreasing that of CFSW (0.69 [0.65–0.74]; Supplementary Figure 10B).

Pregnant women remained the highest-impact population for maximizing QALYs gained under alternative health loss parameter assumptions (Supplementary Figure 11). Diagnostic confirmation among pregnant women averted 3.0% (1.5%–6.2%) of QALY losses under reduced lifetime loss duration assumptions, and 2.6% (1.3%–5.4%) of QALY losses when stillbirths were excluded.

## DISCUSSION

We evaluated allocation of a constrained supply of gonorrhea POCTs to maximize epidemiologic or health impact. Introducing POCTs sufficient to test 0.1% of adults annually achieved the largest morbidity reduction when used for diagnostic confirmation among symptomatic pregnant women (averting 3.5% of QALY losses). Diagnostic confirmation averted the most transmission when delivered among symptomatic FSW (2.1% of incident infections) or their male clients (2.2%), though with smaller morbidity reductions than focusing on pregnant women. Screening asymptomatic clients attending health services was less effective (averting <0.6% of infections or QALY losses) than diagnostic confirmation for all priority populations. With unrestricted POCTs, screening yielded larger absolute effects, but had lower per-test impact than diagnostic confirmation. Although our findings are informed by the Kenyan context, the tradeoff between populations prioritized for transmission versus morbidity reduction is likely relevant to other settings seeking to improve on current syndromic management to address heterosexually-transmitted gonorrhea epidemics.

Limited supplies of POCTs should be prioritized for diagnostic confirmation over screening, supporting WHO guidance to strengthen etiologic diagnosis within syndromic management. Diagnostic confirmation was more efficient because symptomatic care attendees had higher positivity than routine service attendees. With unrestricted tests, screening averted more total infections and QALY losses by detecting asymptomatic infections that would otherwise remain untreated, but it required substantially more tests. Screening may be more beneficial in higher-prevalence populations with sufficient capacity for large-scale targeted testing, as per recent WHO recommendations [7].

Programs aiming to maximize morbidity reduction should prioritize diagnostic confirmation among symptomatic pregnant women, reflecting the high health losses attributed to pregnancy and neonatal sequelae. Testing this group yielded the most health gains per test, despite relatively low infection incidence, as untreated infections in pregnancy caused disproportionate QALY losses. However, focusing exclusively on pregnant women limits broader infection control. In settings with increasing gonorrhea prevalence, strategies targeting incidence reduction may therefore take precedence, whereas in stable or declining epidemics, prioritizing high-burden groups offers greater immediate health gains.

Programs aiming to maximize incidence reduction with constrained POCTs should prioritize diagnostic confirmation among higher-risk populations with symptomatic infection. Testing FSW or their clients was most efficient due to higher prevalence and greater indirect benefits from reduced onward transmission. As POCT availability increases and coverage among high-risk groups saturates, our results suggest that diagnostic confirmation should expand to lower-risk symptomatic males. We evaluated interventions delivered alternately to each population; future modeling should assess combined and sequential test allocation.

Diagnostic confirmation averted more infections among male than female subgroups, despite the already high sensitivity of syndromic management for urethral discharge (80%–90%) [38] compared with the 95% POCT sensitivity for WHO Optimal TPP. The larger impact reflects higher symptom presentation and care-seeking among males, together with a lower proportion of female vaginal discharge syndromic cases attributable to gonorrhea. Anticipated new multiplex POCTs may have higher diagnostic utility for females, since vaginal discharge often has multiple etiologies. As new platforms approach market introduction, their optimal targeting should be re-evaluated.

Screening asymptomatic populations had limited benefit in our model. In lower-prevalence contexts, even highly sensitive and specific POCTs yield low positive predictive values, leading to false-positive diagnoses and unnecessary treatment, which may increase AMR risk. We did not model treatment of false-positive diagnoses or resistance dynamics, as available evidence indicates low current regional cephalosporin resistance [39]. However, intensified screening may drive resistance through greater antibiotic use [40]. Conversely, diagnostic confirmation would reduce overtreatment [13], as syndromic management has poor specificity for both urethral (60%–70%) [38] and vaginal discharge (55%–60%) [8]. These factors support prioritizing POCTs for diagnostic use, especially where overtreatment and antibiotic exposure are common [33]. Future models should incorporate resistance dynamics to align POCT deployment with antimicrobial stewardship.

Data limitations affected both model calibration and parameterization. Population-representative gonorrhea prevalence estimates were scarce, and data for FSW largely came from urban cohort studies. Data gaps necessitated calibration based on male-to-female ratios for men and CFSW, and prevented direct calibration for AGYW and pregnant women. Sexual behavior and care-seeking parameters relied on self-reported data, which are subject to reporting biases [41]. Parameters for CFSW and intermediate risk groups relied on sparse data or assumptions.

Structural assumptions in the transmission model may have influenced intervention impact results. We assumed sexual mixing proportionate to numbers of sexual contacts, which may under-represent the concentration of transmission in younger and higher-risk populations. We also did not incorporate heterogeneity in the frequency of mixing between FSW and CFSW, and did not represent noncommercial partnerships involving FSW. Sexual risk group membership was assumed static over time, which may have over-represented transmission concentration among higher-risk groups. These assumptions are unlikely to have affected prioritization rankings of FSW, CFSW, or pregnant women given large and contrasting differentials between these groups in incidence and morbidity. We excluded post-infection immunity, for which evidence is inconsistent [42–44], enabling rapid reinfection that may have underestimated the impact of POCTs on transmission [44]. We assumed diagnosis depended solely on diagnostic sensitivity and was followed by universal access to effective treatment, overlooking barriers such as stock-outs or informal antibiotic use [45], which may have overestimated the incremental benefit of POCTs over syndromic management. Our modeled screening scenarios did not incorporate delivery constraints, such as outreach to identify CFSW, which could affect the relative costs and feasibility of intervention delivery. We assumed stable baseline gonorrhea prevalence and incidence preintervention, a simplification of longer-term trends but consistent with relatively stable estimates in Eastern Africa [5]. Although biological and behavioral factors suggest AGYW may have higher prevalence than older women, limited and mixed evidence across SSA precluded age differentiation [46–48], potentially underestimating the impact of testing AGYW. Men who have sex with men (MSM) were not included due to our focus on heterosexual transmission and sequelae burden among women, but do have high gonorrhea prevalence in Kenya [49]. Our findings for other high-risk populations suggest prioritizing MSM would be efficient for reducing transmission but less for averting population health losses. Finally, behavioral responses to POCT intervention, such as partner notification, altered care-seeking, or stigma were not modeled, though could affect uptake and effectiveness.

Estimating gonorrhea-related health losses required several assumptions. Sequelae probabilities were primarily derived from chlamydia studies, potentially misrepresenting gonorrhea-specific risks [26]. Several pregnancy-related outcomes, such as premature rupture of membranes and preterm birth, were excluded due to complex interactions with modeled sequelae and insufficient evidence for parameterization [2, 3], potentially underestimating total burden. We assigned stillbirths lifetime QALY losses equivalent to neonatal death, though quantification lacks consensus: some studies exclude stillbirth, while others assign reduced losses [30, 50]. Sensitivity analyses varying stillbirth QALY loss assumptions did not however change prioritization of pregnant women.

In conclusion, gonorrhea POCTs should be deployed for diagnostic confirmation rather than screening when supply is constrained, supporting WHO guidance to strengthen etiologic diagnosis within syndromic management [6]. Programs should prioritize symptomatic pregnant women to maximize morbidity reduction. As new POCTs approach market introduction, particularly those with multiplex or resistance detection capabilities, prioritization strategies should be re-evaluated to reflect diagnostic performance, epidemic dynamics, and the potential for combined targeting approaches.

## Supplementary Material

jiag162_Supplementary_Data

## Supplementary Data

Supplementary materials are available at *The Journal of Infectious Diseases* online (http://jid.oxfordjournals.org/). Supplementary materials consist of data provided by the author that are published to benefit the reader. The posted materials are not copyedited. The contents of all supplementary data are the sole responsibility of the authors. Questions or messages regarding errors should be addressed to the author.

## Notes

***Author contribution.*** A. C., J. M., J. W. I. -E., and M. –C. B. conceptualized the study. J. M. developed the transmission model and probability trees with input from A. C., J. W. I. –E., and M. –C. B. J. M. collated data, conducted literature reviews for model parameterization, and performed all analyses. J. K. and P. B. provided expertise on STI program implementation and epidemiology, informing model development and interpretation of results. J. M. wrote the first draft of the manuscript, which was revised with input from all authors. All authors approved the final manuscript.

***Data availability.*** Data and code underlying this analysis are available at https://doi.org/10.5281/zenodo.18636410.

***Financial support.*** J. M. acknowledges funding from the Imperial College London President’s PhD Fund. A. C., J. M., J. W. I. –E., and M. –C. B. acknowledge funding from the MRC Centre for Global Infectious Disease Analysis (reference MR/R015600/1), jointly funded by the UK Medical Research Council (MRC) and the UK Foreign, Commonwealth & Development Office (FCDO), under the MRC/FCDO Concordat agreement and is also part of the EDCTP2 program supported by the European Union. Under the grant conditions of UKRI, a Creative Commons Attribution 4.0 Generic License (CC BY) has already been assigned to any Author Accepted Manuscript version arising from this submission. The content is solely the responsibility of the authors and does not necessarily represent the official views of the funders.
