# Supplementary material for: Optimal Deployment of Gonorrhoea Point-of-Care Tests: Modeling the Potential Impact of Diagnostic Confirmation Testing and Screening Strategies Across Five Priority Populations in Kenya
Source: J Infect Dis. 2026 Mar 17;234(3):e714–23. doi: 10.1093/infdis/jiag162 (PMC13600434; doi:10.1093/infdis/jiag162)
Supplement: Supplementary file 1 [file jiag162_supplementary_data.pdf]

Optimal deployment of gonorrhoea point-of-care tests: modelling the potential impact of diagnostic confirmation testing and screening strategies across five priority populations in Kenya

Supplementary material

Julia Michalow[1*], Anne Cori[1], Joshua Kimani[2], Parinita Bhattacharjee[2,3], Prof Marie-Claude Boily[1], Jeffrey W. Imai-Eaton[1,3]

[1] MRC Centre for Global Infectious Disease Analysis, School of Public Health, Imperial College London, London, United Kingdom

[2] Partners for Health and Development in Africa (PHDA), Nairobi, Kenya

[3] Institute for Global Public Health, University of Manitoba, Winnipeg, Manitoba, Canada

[4] Center for Communicable Disease Dynamics, Department of Epidemiology, Harvard T.H. Chan School of Public Health, Boston, MA, USA

[*] Corresponding author: Julia Michalow (j.michalow21@imperial.ac.uk)

Contents

1 Supplementary methods **1**

1.1 Transmission model details 1

1.2 Transmission model parametrisation 8

1.3 Transmission model calibration 13

1.4 Quality-adjusted life years lost 17

2 Supplementary results **24**

2.1 Transmission model calibration 24

2.2 Baseline outcomes 28

2.3 Intervention outcomes 31

2.4 Sensitivity analyses 33

Bibliography **36**

List of Figures

S1 Flow diagram of gonorrhoea transmission model 2
S2 Estimated prevalence of gonorrhoea among the general population and female sex workers in Kenya, 2000-2025 14
S3 Probability trees for gonorrhoea sequelae 17
S4 Comparison of model population estimates with UN WPP projections 24
S5 Distribution of baseline model outcomes after calibration 25
S6 Prior and posterior distributions for calibrated parameters 26
S7 Rank correlations among posterior samples for calibrated model parameters 27
S8 Distribution of incident gonorrhoea infections by symptom and treatment status at baseline, 2025 29
S9 Decomposition of total discounted lifetime QALYs lost 30
S10 Correlation between parameter values and priority population prioritisation order by test strategy 33
S10 Correlation between parameter values and priority population prioritisation order by test strategy (continued) 34
S11 Influence of parameter assumptions for pregnancy-related sequelae on the predicted population-level impact of diagnostic confirmation and screening strategies, 2025–2030 35

List of Tables

S1 Demographic parameter values 9

S2 Parameter definitions, priors, and posteriors for transmission model 11

S3 Calibration outcomes 13

S4 Study observations of gonorrhoea prevalence in Kenya 15

S5 Parameters for the probability, duration, and utility of gonorrhoea sequelae 19

S6 Probability of adverse outcomes in pregnant women with untreated gonorrhoea. 21

S7 Years lived with health losses due to each sequela 23

S8 Predicted gonorrhoea burden at baseline, 2025 28

S9 Predicted population-level impact of diagnostic confirmation testing and screening strategies among each priority population with constrained POCT availability, 2025–2030 31

S10 Predicted population-level impact of diagnostic confirmation testing and screening strategies among each priority population with unrestricted POCT availability, 2025–2030 32

1 Supplementary methods

1.1 Transmission model details

The modelled population was stratified by sex (male and female), sexual risk (low, intermediate, and high, with high representing female sex workers [FSWs] and clients of female sex workers [CFSWs]), age group (15-24 years [younger] and 25-49 years [older]), and gestational status (non-pregnant and pregnant), denoted as $s \in \{m, f\}$, $r \in \{l, i, h\}$, $a \in \{y, o\}$, $g \in \{n, p\}$ throughout this supplement. Age groups were selected to align with the HIV/STI programme definition for adolescent girls and young women (AGYW; 15–24 years).[1]

1.1.1 Equations

For an individual of a given sex (s), sexual risk (r), age (a), and gestational status (g), the model is described by the following system of differential equations. In these equations, stratification indices are represented numerically as: $s \in \{0, 1\}$ for male and female; $r \in \{0, 1, 2\}$ for low, intermediate, and high sexual risk; $a \in \{0, 1\}$ for 15–24 and 25–49 years; and $g \in \{0, 1\}$ for non-pregnant and pregnant.

Parameters are defined in Table S2. In Equation 1.1, there are no terms relating to pregnancy (f_{ra} and α) as individuals have not yet become sexually active. In Equations 1.6 - 1.10, intervention-related parameters (δ_{srag} or τ^{h}_{srag}) are set to zero in scenarios where diagnostic confirmation or screening interventions are not active. This allows all compartments and transitions to be retained in the model system while isolating intervention effects analytically. Intervention scenarios are detailed in Text 1.1.4.

In this chapter, $\mathbb{1}_A$ is used as an indicator function such that:

$$\mathbb{1}_A := \begin{cases} 1 & \text{if } A \text{ is true} \\ 0 & \text{if } A \text{ is false} \end{cases}$$

$$\frac{dU_{s,r,a,g}(t)}{dt} = ent_{s,r,a,g} - \psi_{s,r,a,g}\, U_{s,r,a,g} - \omega_a\, U_{s,r,a,g} + \mathbb{1}_{\{a=1\}}\, \omega_{a-1}\, U_{s,r,a-1,g} - \mu_{s,a}\, U_{s,r,a,g} \tag{1.1}$$

$$\begin{aligned} \frac{dS_{s,r,a,g}(t)}{dt} &= \psi_{s,r,a,g}\, U_{s,r,a,g} - \lambda_{s,r,a,g}\, S_{s,r,a,g} \\ &+ \pi(X_{s,r,a,g} + Y_{s,r,a,g} + Z_{s,r,a,g} + R^{y}_{s,r,a,g} + R^{h}_{s,r,a,g}) + \sigma(M^{y}_{s,r,a,g} + T^{y}_{s,r,a,g} + T^{h}_{s,r,a,g}) \\ &- \mathbb{1}_{\{s=1,g=0\}}\, f_{ra}\, S_{s,r,a,g} + \mathbb{1}_{\{s=1,g=1\}}\, f_{ra}\, S_{s,r,a,g-1} + \mathbb{1}_{\{s=1,g=0\}}\, \alpha\, S_{s,r,a,g+1} - \mathbb{1}_{\{s=1,g=1\}}\, \alpha\, S_{s,r,a,g} \\ &- \omega_a\, S_{s,r,a,g} + \mathbb{1}_{\{a=1\}}\, \omega_{a-1}\, S_{s,r,a-1,g} - \mu_{s,a}\, S_{s,r,a,g} \end{aligned} \tag{1.2}$$

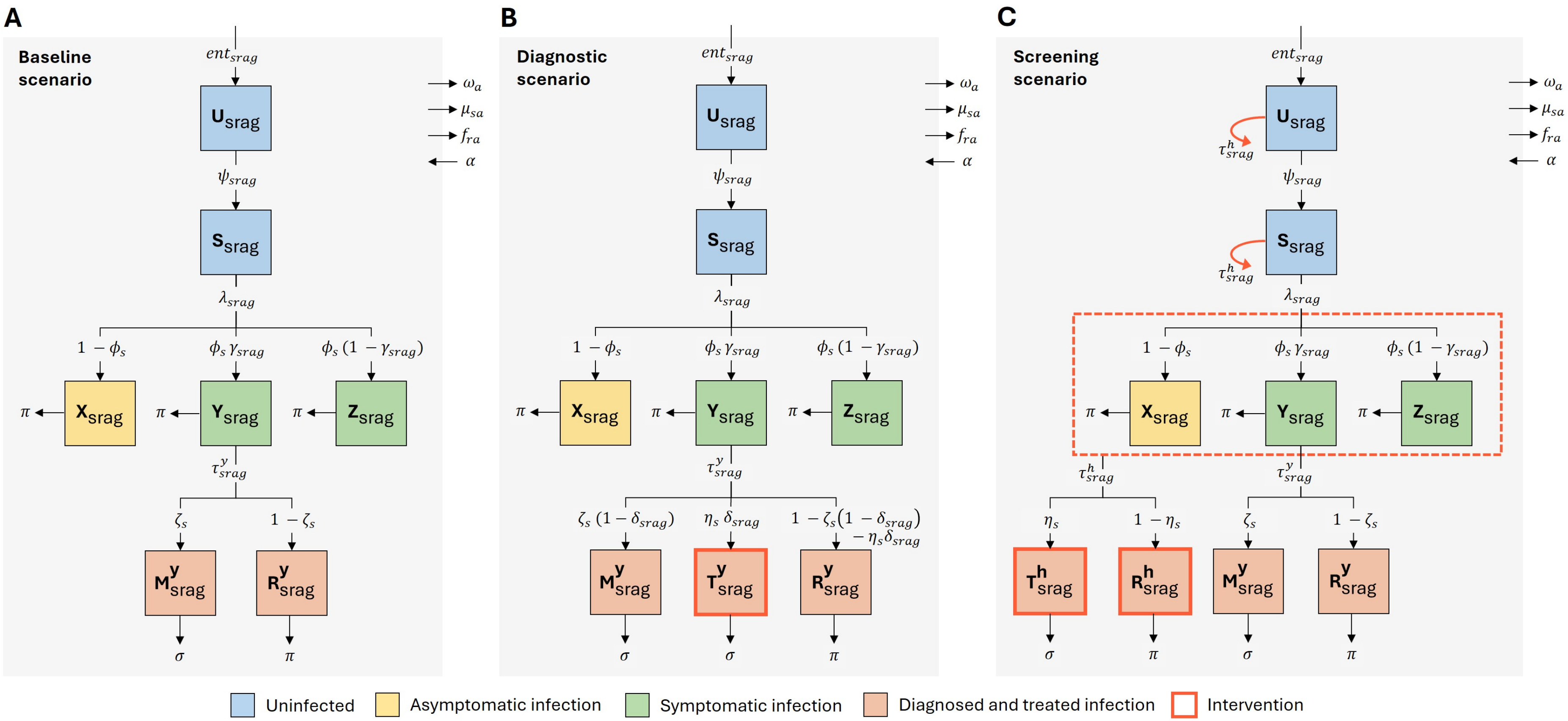

Figure S1: Flow diagram of gonorrhoea transmission model

Model structure for diagnostic and treatment scenarios: (A) baseline with syndromic management, (B) diagnostic confirmation testing, and (C) screening. Population states shown as boxes: blue for uninfected (U: never sexually active, S: susceptible), yellow for asymptomatic infection (X), green for symptomatic infection (Y: with healthcare access, Z: without), and orange for diagnosed and treated infection (M^y: appropriate treatment after syndromic management, T^y or T^h: appropriate treatment following POCT for diagnosis or screening, R^y or R^h: inappropriate treatment). Dark orange boxes and arrows indicate POCT intervention components; dotted box in (C) indicates application to all three infection compartments. Subscripts denote stratification by sex (s), risk (r), age (a), and gestational status (g). Superscripts denote treatment among symptomatic individuals (y) or routine healthcare attendees (h). Transitions include rates for becoming sexually active (ψ), force of infection (λ), treatment among symptomatics (τ^y), screening with POCT (τ^h), recovery after treatment (σ), and natural recovery (π). Recovered individuals return to susceptible state (arrows omitted for diagrammatic clarity). Parameters define probabilities of symptomatic infection (ϕ), accessing care among symptomatics (γ), syndromic management sensitivity (ζ), POCT diagnosis among symptomatics (δ), and POCT sensitivity (η). Outflows represent ageing (ω), mortality (μ), pregnancy (f), or return from pregnancy (α) for relevant compartment strata. Table S2 contains detailed parameter definitions.

$$\begin{aligned}\frac{dX_{s,r,a,g}(t)}{dt} &= \lambda_{s,r,a,g}\ (1-\phi_s)\ S_{s,r,a,g} - \pi\ X_{s,r,a,g} - \tau^h_{s,r,a,g}\ X_{s,r,a,g} \\ &- \mathbb{1}_{\{s=1,g=0\}}\ f_{ra}\ X_{s,r,a,g} + \mathbb{1}_{\{s=1,g=1\}}\ f_{ra}\ X_{s,r,a,g-1} + \mathbb{1}_{\{s=1,g=0\}}\ \alpha\ X_{s,r,a,g+1} - \mathbb{1}_{\{s=1,g=1\}}\ \alpha\ X_{s,r,a,g} \\ &- \omega_a\ X_{s,r,a,g} + \mathbb{1}_{\{a=1\}}\ \omega_{a-1}\ X_{s,r,a-1,g} - \mu_{s,a}\ X_{s,r,a,g}\end{aligned} \tag{1.3}$$

$$\begin{aligned}\frac{dY_{s,r,a,g}(t)}{dt} &= \lambda_{s,r,a,g}\ \phi_s\ \gamma_{s,r,a,g}\ S_{s,r,a,g} - \pi\ Y_{s,r,a,g} - \tau^y_{s,r,a,g}\ Y_{s,r,a,g} - \tau^h_{s,r,a,g}\ Y_{s,r,a,g} \\ &- \mathbb{1}_{\{s=1,g=0\}}\ f_{ra}\ Y_{s,r,a,g} + \mathbb{1}_{\{s=1,g=1\}}\ f_{ra}\ Y_{s,r,a,g-1} + \mathbb{1}_{\{s=1,g=0\}}\ \alpha\ Y_{s,r,a,g+1} - \mathbb{1}_{\{s=1,g=1\}}\ \alpha\ Y_{s,r,a,g} \\ &- \omega_a\ Y_{s,r,a,g} + \mathbb{1}_{\{a=1\}}\ \omega_{a-1}\ Y_{s,r,a-1,g} - \mu_{s,a}\ Y_{s,r,a,g}\end{aligned} \tag{1.4}$$

$$\begin{aligned}\frac{dZ_{s,r,a,g}(t)}{dt} &= \lambda_{s,r,a,g}\ \phi_s\ (1-\gamma_{s,r,a,g})\ S_{s,r,a,g} - \pi\ Z_{s,r,a,g} - \tau^h_{s,r,a,g}\ Z_{s,r,a,g} \\ &- \mathbb{1}_{\{s=1,g=0\}}\ f_{ra}\ Z_{s,r,a,g} + \mathbb{1}_{\{s=1,g=1\}}\ f_{ra}\ Z_{s,r,a,g-1} + \mathbb{1}_{\{s=1,g=0\}}\ \alpha\ Z_{s,r,a,g+1} - \mathbb{1}_{\{s=1,g=1\}}\ \alpha\ Z_{s,r,a,g} \\ &- \omega_a\ Z_{s,r,a,g} + \mathbb{1}_{\{a=1\}}\ \omega_{a-1}\ Z_{s,r,a-1,g} - \mu_{s,a}\ Z_{s,r,a,g}\end{aligned} \tag{1.5}$$

$$\begin{aligned}\frac{dM^y_{s,r,a,g}(t)}{dt} &= \tau^y_{s,r,a,g}\ \zeta_s\ (1-\delta_{s,r,a,g})\ Y_{s,r,a,g} - \sigma\ M^y_{s,r,a,g} \\ &- \mathbb{1}_{\{s=1,g=0\}}\ f_{ra}\ M^y_{s,r,a,g} + \mathbb{1}_{\{s=1,g=1\}}\ f_{ra}\ M^y_{s,r,a,g-1} + \mathbb{1}_{\{s=1,g=0\}}\ \alpha\ M^y_{s,r,a,g+1} - \mathbb{1}_{\{s=1,g=1\}}\ \alpha\ M^y_{s,r,a,g} \\ &- \omega_a\ M^y_{s,r,a,g} + \mathbb{1}_{\{a=1\}}\ \omega_{a-1}\ M^y_{s,r,a-1,g} - \mu_{s,a}\ M^y_{s,r,a,g}\end{aligned} \tag{1.6}$$

$$\begin{aligned}\frac{dT^y_{s,r,a,g}(t)}{dt} &= \tau^y_{s,r,a,g}\ \eta_s\ \delta_{s,r,a,g}\ Y_{s,r,a,g} - \sigma\ T^y_{s,r,a,g} \\ &- \mathbb{1}_{\{s=1,g=0\}}\ f_{ra}\ T^y_{s,r,a,g} + \mathbb{1}_{\{s=1,g=1\}}\ f_{ra}\ T^y_{s,r,a,g-1} + \mathbb{1}_{\{s=1,g=0\}}\ \alpha\ T^y_{s,r,a,g+1} - \mathbb{1}_{\{s=1,g=1\}}\ \alpha\ T^y_{s,r,a,g} \\ &- \omega_a\ T^y_{s,r,a,g} + \mathbb{1}_{\{a=1\}}\ \omega_{a-1}\ T^y_{s,r,a-1,g} - \mu_{s,a}\ T^y_{s,r,a,g}\end{aligned} \tag{1.7}$$

$$\begin{aligned}\frac{dR^y_{s,r,a,g}(t)}{dt} &= \tau^y_{s,r,a,g}\ [1-\zeta_s(1-\delta_{s,r,a,g}) - \eta_s\delta_{s,r,a,g}]\ Y_{s,r,a,g} - \pi\ R^y_{s,r,a,g} \\ &- \mathbb{1}_{\{s=1,g=0\}}\ f_{ra}\ R^y_{s,r,a,g} + \mathbb{1}_{\{s=1,g=1\}}\ f_{ra}\ R^y_{s,r,a,g-1} + \mathbb{1}_{\{s=1,g=0\}}\ \alpha\ R^y_{s,r,a,g+1} - \mathbb{1}_{\{s=1,g=1\}}\ \alpha\ R^y_{s,r,a,g} \\ &- \omega_a\ R^y_{s,r,a,g} + \mathbb{1}_{\{a=1\}}\ \omega_{a-1}\ R^y_{s,r,a-1,g} - \mu_{s,a}\ R^y_{s,r,a,g}\end{aligned} \tag{1.8}$$

$$\begin{aligned}\frac{dT^h_{s,r,a,g}(t)}{dt} &= \tau^h_{s,r,a,g}\ \eta_s\ (X_{s,r,a,g} + Y_{s,r,a,g} + Z_{s,r,a,g}) - \sigma\ T^h_{s,r,a,g} \\ &- \mathbb{1}_{\{s=1,g=0\}}\ f_{ra}\ T^h_{s,r,a,g} + \mathbb{1}_{\{s=1,g=1\}}\ f_{ra}\ T^h_{s,r,a,g-1} + \mathbb{1}_{\{s=1,g=0\}}\ \alpha\ T^h_{s,r,a,g+1} - \mathbb{1}_{\{s=1,g=1\}}\ \alpha\ T^h_{s,r,a,g} \\ &- \omega_a\ T^h_{s,r,a,g} + \mathbb{1}_{\{a=1\}}\ \omega_{a-1}\ T^h_{s,r,a-1,g} - \mu_{s,a}\ T^h_{s,r,a,g}\end{aligned} \tag{1.9}$$

$$\begin{aligned}\frac{dR^h_{s,r,a,g}(t)}{dt} &= \tau^h_{s,r,a,g}\ (1-\eta_s)\ (X_{s,r,a,g} + Y_{s,r,a,g} + Z_{s,r,a,g}) - \pi\ R^h_{s,r,a,g} \\ &- \mathbb{1}_{\{s=1,g=0\}}\ f_{ra}\ R^h_{s,r,a,g} + \mathbb{1}_{\{s=1,g=1\}}\ f_{ra}\ R^h_{s,r,a,g-1} + \mathbb{1}_{\{s=1,g=0\}}\ \alpha\ R^h_{s,r,a,g+1} - \mathbb{1}_{\{s=1,g=1\}}\ \alpha\ R^h_{s,r,a,g} \\ &- \omega_a\ R^h_{s,r,a,g} + \mathbb{1}_{\{a=1\}}\ \omega_{a-1}\ R^h_{s,r,a-1,g} - \mu_{s,a}\ R^h_{s,r,a,g}\end{aligned} \tag{1.10}$$

The total (N), sexually active ($\hat{N}$), and infected (I) population are calculated as:

$$N_{s,r,a,g} = U_{s,r,a,g} + S_{s,r,a,g} + I_{s,r,a,g} \tag{1.11}$$

$$\hat{N}_{s,r,a,g} = S_{s,r,a,g} + I_{s,r,a,g} \tag{1.12}$$

$$I_{s,r,a,g} = X_{s,r,a,g} + Y_{s,r,a,g} + Z_{s,r,a,g} + M^y_{s,r,a,g} + T^y_{s,r,a,g} + R^y_{s,r,a,g} + T^h_{s,r,a,g} + R^h_{s,r,a,g} \tag{1.13}$$

1.1.2 Sexual mixing

Sexual mixing was implemented as fully proportionate. Partner acquisition was assumed to be independent of infection status, such that individuals acquired sexual partners at the same rate regardless of whether they were uninfected, asymptomatically infected, or symptomatically infected. The mixing matrix ($\rho_{srags'r'a'g'}$) represented the probability that an individual of type s, r, a, g selects a sexual partner of type s', r', a', g', conditional on forming a new partnership. This probability was proportional to the total number of partnerships offered by the opposite sex, as determined by their sexually active population size ($\hat{N}_{s'r'a'g'}$) and their average annual rate of partner acquisition ($c_{s'r'a'g'}$). The mixing matrix also incorporated structural constraints in the sexual network ($\theta_{srags'r'a'g'}$): (1) partnerships were only possible between individuals of the opposite sex, and (2) FSW formed partnerships exclusively with their clients (high-risk males), while clients could form partnerships with females from all risk groups.

$$\rho_{srags'r'a'g'} = \frac{\theta_{srags'r'a'g'}\ c_{s'r'a'g'}\ \hat{N}_{s'r'a'g'}}{\sum_{r''=l}^{h} \sum_{a''=y}^{o} \sum_{g''=n}^{p} \theta_{srags''r''a''g''}\ c_{s''r''a''g''}\ \hat{N}_{s''r''a''g''}} \tag{1.14}$$

$$\theta_{srags'r'a'g'} = \begin{cases} 0 & \text{Same-sex partnerships} \\ & \quad s = s' \\ 0 & \text{FSW and non-client partnerships} \\ & \quad s = f,\ r = h,\ s' = m,\ r' \in \{l, i\} \\ & \quad s = m,\ r \in \{l, i\},\ s' = f,\ r' = h \\ 1 & \text{Other partnerships} \end{cases} \tag{1.15}$$

To ensure consistency in the number of partnerships formed between sexes, a balancing procedure was applied to the annual rates of partner acquisition,[2] since reported sexual partner numbers often differ between men and women. A balancing factor ($B_{srags'r'a'g'}$) quantified the degree of imbalance in the total number of partnerships formed by individuals of type s, r, a, g with those of type $s'r'a'g$:

$$B_{srags'r'a'g'} = \frac{c_{srag}\ \rho_{srags'r'a'g'}\ \hat{N}_{srag}}{c_{s'r'a'g'}\ \rho_{s'r'a'g'srag}\ \hat{N}_{s'r'a'g'}} \tag{1.16}$$

A value of $B_{srags'r'a'g'} = 1$ indicated perfect balance, while values greater or less than 1 indicated that individuals of sex s are forming more or fewer partnerships with individuals of sex s', respectively. To preserve relative differences in partner acquisition across groups while ensuring symmetry in the total number of partnerships formed between any two strata, the initial rates were adjusted as follows:

$$c^*_{srags'r'a'g'} = c_{srag}\ B^{-0.5}_{srags'r'a'g'} \tag{1.17}$$

$$c^*_{s'r'a'g'srag} = c_{s'r'a'g'}\ B^{0.5}_{srags'r'a'g'} \tag{1.18}$$

The adjusted rate ($c^*_{srags'r'a'g'}$) represented the effective annual rate at which individuals of type s, r, a, g acquired new partners of type s', r', a', g', after accounting for imbalances in partnership numbers between sexes. The product $c^*_{srags'r'a'g'}\rho_{srags'r'a'g'}$ therefore reflected the annual number of new partners of type s', r', a', g' acquired by individuals of type s, r, a, g.

1.1.3 Force of infection

The force of infection (λ_{srag}) determined the rate per year at which a susceptible individual of type s, r, a, g acquired infection through the formation of new sexual partnerships. This depended on the per-partnership probability of transmission ($\kappa^p_{srags'r'a'g'}$), the adjusted rate of acquiring new partners ($c^*_{srags'r'a'g'}$), the probability of selecting such a partner conditional on partnership formation ($\rho_{srags'r'a'g'}$), and the prevalence of infection among potential partners ($I_{s'r'a'g'}/\hat{N}_{s'r'a'g'}$):

$$\lambda_{srag} = \sum_{r'=l}^{h} \sum_{a'=y}^{o} \sum_{g'=n}^{p} \kappa^p_{srags'r'a'g'}\ c^*_{srags'r'a'g'}\ \rho_{srags'r'a'g'}\ \frac{I_{s'r'a'g'}}{\hat{N}_{s'r'a'g'}} \tag{1.19}$$

The per-partnership transmission probability ($\kappa^p_{srags'r'a'g'}$) was determined based on the transmission probability per sex act (κ^a_s), the number of sex acts per partnership ($n^p_{srags'r'a'g'}$), the proportion of sex acts within the partnership where a condom is used ($\chi^p_{srags'r'a'g'}$), and the efficacy of condoms in preventing transmission (e). The formulation considered the probability of avoiding infection across all sex acts within the partnership, accounting separately for acts with and without condom use:

$$\kappa^p_{srags'r'a'g'} = 1\ -\ (1-(1-e)\kappa^a_s)^{n^p_{srags'r'a'g'}\chi^p_{srags'r'a'g'}}\ (1-\kappa^a_s)^{n^p_{srags'r'a'g'}(1-\chi^p_{srags'r'a'g'})} \tag{1.20}$$

Since data were lacking to quantify the number of sex acts within each partnership type, it was assumed that the annual number of sex acts per individual (n) was the same across all strata, and that sex acts were distributed equally across partners. FSW and their clients were assumed to have only one sex act per partnership. The number of sex acts per partnership was therefore defined as:

$$n^p_{srags'r'a'g'} = \begin{cases} 1 & \text{FSW and client partnerships} \\ \frac{n}{c^*_{srags'r'a'g'} \rho_{srags'r'a'g'}} & \text{Other partnerships} \end{cases} \tag{1.21}$$

To ensure consistency in condom use assumptions across partner types, the proportion of sex acts within each partnership that used a condom ($\chi^p_{srags'r'a'g'}$) was calculated by equally weighting each partner's reported condom use at last sex:

$$\chi^p_{srags'r'a'g'} = (\chi_{srag} + \chi_{s'r'a'g'})^{0.5} \tag{1.22}$$

1.1.4 Intervention scenarios

Point-of-care testing for gonorrhoea was implemented from 2025 to 2030. Tests were allocated to one of five priority population groups: pregnant women (all ages and risk groups); sexually active adolescent girls and young women (AGYW; non-pregnant 15-24-year-old females who had ever had sex); FSW (all ages and gestational status); the total male population (all ages and risk groups); and CFSW (high-risk males of all ages). For each priority population, tests were used as either a diagnostic confirmation or screening intervention.

Diagnostic confirmation: In the diagnostic confirmation scenario, POCTs were implemented among individuals accessing care for urethral discharge and vaginal discharge syndromes. The eligible population was determined as the annual number of symptomatic cases caused by gonorrhoea (NG-case$_{srag}$) and other reproductive tract infections (RTI-case$_{srag}$), such that the same individual could be tested multiple times if they experienced repeat infections.

The number of symptomatic gonorrhoea cases accessing treatment per year (y) was defined as the total number of individuals leaving the Y compartment, where Y_{srag} represented symptomatic gonorrhoea infections in individuals planning to access treatment. This was calculated as:

$$\text{NG-case}_{srag}(y) = \int_{t=y}^{y+1} \tau^y_{srag}\, Y_{srag}(t)\, dt \tag{1.23}$$

Cases due to other RTIs were estimated to define the pool of symptomatic care attendees eligible

for diagnostic testing and were not modelled as transmissible infections. Therefore, the model did not capture incidental diagnosis and treatment of gonorrhoea among individuals accessing care for symptoms caused by a concurrent non-gonococcal RTI. Annual symptomatic cases due to other RTIs were calculated using the gonorrhoea aetiologic proportion (p_s), defined as the proportion of individuals with a given symptom who have gonorrhoea as the underlying cause. This allowed estimation of total symptomatic cases attributable to causes other than gonorrhoea by scaling observed symptomatic gonorrhoea cases accordingly. These RTI case numbers were then fixed at pre-intervention levels to ensure they remained independent of changes in gonorrhoea incidence during intervention simulations:

$$\text{RTI-case}_{srag}(y) = \begin{cases} \text{NG-case}_{srag}(y)\ \frac{(1-p_s)}{p_s} & y < 2025 \\ \text{NG-case}_{srag}(y)\ \frac{(1-p_s)}{p_s}\Big|_{y=2024} & y \geq 2025 \end{cases} \tag{1.24}$$

The annual probability of receiving a diagnostic confirmation test within a selected priority population (δ_T) was calculated as the ratio of available tests to the total number of eligible symptomatic cases in that population. Each priority population T comprised a subset of strata defined by sex, risk, age, and gestational status, such that $(srag) \in T$ denoted the included combinations. A maximum probability of 1 was imposed to ensure that testing coverage did not exceed the number of eligible individuals:

$$\delta_T = \min\left(1, \frac{\text{annual tests available for } T}{\sum_{(srag)\in T} \text{NG-case}_{srag}(y) + \text{RTI-case}_{srag}(y)}\right) \tag{1.25}$$

Screening: In the screening scenario, POCTs were implemented among individuals attending either primary healthcare or antenatal care services. The eligible population was defined as the number of individuals accessing these health services annually. Since individuals may attend care more than once per year, the annual probability of healthcare attendance (hc_{srag}) was converted to a rate, assuming visits follow a Poisson process with exponential spacing over time. The effective number of individuals attending healthcare services annually and eligible for screening was calculated as:

$$N^{\text{h}}_{srag} = -\log(1 - \text{hc}_{srag})(U_{srag} + S_{srag} + X_{srag} + Y_{srag} + Z_{srag}) \tag{1.26}$$

For each priority population T, the annual screening rate (τ^{h}_T) was calculated as the ratio of available tests to the total number of eligible individuals in the priority population:

$$\tau^{\text{h}}_T = \frac{\text{annual tests available for } T}{\sum_{(srag)\in T} N^{\text{h}}_{srag}} \tag{1.27}$$

The same annual screening rate was applied to each stratum $srag \in T$.

1.2 Transmission model parametrisation

1.2.1 Demographic parameters

Population size was derived using age- and sex-stratified demographic estimates for the 15-49-year-old Kenyan population between 1970 and 2030 from UN World Population Prospects (WPP) 2024.[3] The female population was further stratified by gestational status. The number of pregnant women in year y was estimated according to the number of births by maternal age group in the following year (B_{fa}) and the annual rate of delivery (α), corresponding to a mean gestational period of 9 months (i.e. $1/\alpha = 0.75$ year):

$$N_{fap}(y) = \alpha B_{fa}(y+1) \tag{1.28}$$

Annual pregnancy rates for females were then calculated per age group as the ratio of pregnant to non-pregnant women in that group, scaled by α to account for pregnancy duration:

$$f_a = \alpha \frac{N_{fap}}{N_{fan}} \tag{1.29}$$

To estimate annual pregnancy rates among sexually active females, population-level rates were adjusted by the proportion sexually active in each non-pregnant female stratum (q_{fran}), which varied by risk group, such that:

$$f_{ra}^{model} = \frac{f_a}{q_{fran}} \tag{1.30}$$

This adjustment ensured that the total number of pregnancies was preserved. If all non-pregnant females in a stratum were sexually active ($q_{fran} = 1$), no adjustment was needed. If only a subset were sexually active ($q_{fran} < 1$), the per-capita pregnancy rate among those individuals was increased proportionally.

Each sex-age-gestational stratum was divided into sexual risk groups according to the sex-specific probability of belonging to a risk group (υ_{sr}):

$$N_{srag}(y) = \upsilon_{sr} N_{sag}(y) \tag{1.31}$$

New male and female entrants to the population were aged 15-24 years and allocated to the U compartment. The annual number of entrants was calculated using sex-stratified births 15 years prior (B_s) and deaths in corresponding sex and age cohorts (D_s^{age}) at the midpoint of exposure.[3] All entrants

were non-pregnant and were stratified by risk group using the same method as for the total population.

$$ent_{srag}(y) = \mathbb{1}_{\{a=15-24,g=n\}}\, \upsilon_{sr}\left[B_s(y-15) - D_s^{0-4}(y-13) - D_s^{5-9}(y-8) - D_s^{10-14}(y-3)\right] \qquad (1.32)$$

Values were calculated for 1970 and 2030, with intermediate years interpolated linearly.

Individuals aged from 15–24 to 25–49 years and exited the 25-49 year-old group at the annual rate ω_a. Sex and risk group were assumed to remain fixed over time, while gestational status changed dynamically for females based on the age- and risk-specific pregnancy rates described above.

Mortality rates (μ_{sa}) from all causes were calculated by dividing total deaths by population size per sex and age in 1970 and 2030, with values interpolated linearly for intermediate years.[3]

Table S1: Demographic parameter values

**Stratum**	**Population**		**Entrants**		**Pregnancy rate**		**Mortality rate**	
	1970	2030	1970	2030	1970	2030	1970	2030
m.l.y.n	730 072	4 089 571	96 749	485 610	0	0	0.0042	0.0020
m.l.y.p	0	0	0	0	0	0	0	0
m.l.o.n	729 196	6 017 108	0	0	0	0	0.0073	0.0060
m.l.o.p	0	0	0	0	0	0	0	0
m.i.y.n	157 253	880 867	20 839	104 597	0	0	0.0042	0.0020
m.i.y.p	0	0	0	0	0	0	0	0
m.i.o.n	157 064	1 296 046	0	0	0	0	0.0073	0.0060
m.i.o.p	0	0	0	0	0	0	0	0
m.h.y.n	154 455	865 197	20 468	102 736	0	0	0.0042	0.0020
m.h.y.p	0	0	0	0	0	0	0	0
m.h.o.n	154 270	1 272 990	0	0	0	0	0.0073	0.0060
m.h.o.p	0	0	0	0	0	0	0	0
f.l.y.n	607 808	3 870 978	101 486	497 481	0.49	0.19	0.0036	0.0015
f.l.y.p	132 960	339 634	0	0	0	0	0.0036	0.0015
f.l.o.n	735 096	5 828 462	0	0	0.32	0.11	0.0062	0.0043
f.l.o.p	177 531	487 227	0	0	0	0	0.0062	0.0043
f.i.y.n	208 485	1 332 056	34 811	170 642	0.45	0.17	0.0036	0.0015
f.i.y.p	45 607	112 233	0	0	0	0	0.0036	0.0015
f.i.o.n	252 147	1 999 513	0	0	0.32	0.11	0.0062	0.0043
f.i.o.p	60 895	166 843	0	0	0	0	0.0062	0.0043
f.h.y.n	16 827	107 853	2 810	13 773	0.41	0.16	0.0036	0.0015
f.h.y.p	3 681	8 717	0	0	0	0	0.0036	0.0015
f.h.o.n	20 351	161 406	0	0	0.32	0.11	0.0062	0.0043
f.h.o.p	4 915	13 443	0	0	0	0	0.0062	0.0043

Parameters derived using UN WPP 2024 estimates.[3] Values shown use median posterior estimates for the probability of being in a sexual risk group (υ_{sr}) and the probability of being sexually active (q_{srag}).

1.2.2 Sexual behaviour, diagnosis, and treatment parameters

Parameter values related to sexual behaviour and care-seeking for symptoms were derived from Kenya Demographic and Health Surveys (DHS) in 2003, 2008-2009, 2014 and 2022,[4–7] and Kenya Population-based HIV Impact Assessment (PHIA) in 2018.[8] For the parameters listed below, values were primarily estimated by pooling DHS and summarising data as survey-weighted proportions with design-based standard errors and 95% confidence intervals. The PHIA covered fewer relevant indicators; data were extracted from the published report as supplementary input. Variables included:

- Percentage of males who had ever paid for sex.[4,6]
- Percentage of adults who had ever had sex, stratified by sex and age group.[4–7]
- Number of sexual partners reported among sexually active adults in the past year, stratified by sex, age group, and gestational status.[4–7]
- Percentage of sexually active adults reporting condom use at last sex, stratified by sex, age group,[4–8] gestational status,[4–7] marital status,[4–8] and higher-risk sex.[4–7]
- Percentage of sexually active adults with genital discharge who reported accessing care in the past year, stratified by sex, age group, and pregnancy status.[4–7]
- Percentage of women who attended ANC at least once for their most recent pregnancy.[4–7]

Table S2: Parameter definitions, priors, and posteriors for transmission model

Description	Symbol	Prior Distribution	Posterior, Median (UI)	Source
**Demography**				
Initial population size in 1970	N^0_{srag}	Fixed	Table S1	[3]
Annual entrants into population	ent_{srag}	Fixed	Table S1	[3]
Probability of being in sexual risk group				
Male, low risk	υ_{ml}	Calculated	0.70 (0.60-0.80)	$1 - \upsilon_{mi} - \upsilon_{mh}$
Male, intermediate risk	υ_{mi}	U(0.10, 0.20)	0.15 (0.10-0.20)	Assumption
Male, high risk (CFSW)	υ_{mh}	U(0.10, 0.20)	0.15 (0.10-0.20)	[4–7]
Female, low risk	υ_{fl}	Calculated	0.73 (0.67-0.79)	$1 - \upsilon_{fi} - \upsilon_{fh}$
Female, intermediate risk	υ_{fi}	U(0.20, 0.30)	0.25 (0.20-0.30)	Assumption
Female, high risk (FSW)	υ_{fh}	U(0.01, 0.03)	0.02 (0.01-0.03)	[9]
Mean duration in age group (year)				
15-24 y	$1/\omega_y$	Fixed	10	-
25-49 y	$1/\omega_o$	Fixed	25	-
Rate of becoming pregnant among sexually active females (year^{-1})	f_{ra}	Fixed	Table S1	[3]
Mean duration of pregnancy (year)	$1/\alpha$	Fixed	0.75	-
Rate of mortality (year^{-1})	μ_{sa}	Fixed	Table S1	[3]
**Sexual behaviour**				
Probability of being sexually active				
All sex, low risk, 15-24 y, non-pregnant	q_{slyn}	U(0.50, 0.70)	0.59 (0.50-0.70)	[4–7]
All sex, all risk, 25-49 y, non-pregnant	q_{sron}	Fixed	1	[4–7]
Female, all risk, all age, pregnant	q_{frap}	Fixed	1	-
Relative probability of being sexually active among all sex, 15-24 y, non-pregnant				
Intermediate:low risk	$rq_{i:l}$	U(1.05, 1.15)	1.10 (1.05-1.15)	Assumption
High:intermediate risk	$rq_{h:i}$	U(1.05, 1.15)	1.10 (1.05-1.15)	Assumption
Rate of becoming sexually active (year^{-1})	ψ_{srag}	Calculated	-	$-log(1 - q_{srag})$
Number of partnerships per person (year^{-1})				
All sex, low risk, 15-24 y, non-pregnant	c_{slyn}	U(1.00, 1.50)	1.24 (1.01-1.48)	[4–7]
Relative number of partnerships per person				
Male, intermediate:low risk	$rc_{mi:ml}$	U(3.00, 6.00)	4.20 (3.08-5.87)	Assumption
Male, high:intermediate risk	$rc_{mh:mi}$	U(2.50, 5.00)	2.84 (2.05-3.89)	[4,6]
Female, intermediate:low risk	$rc_{fi:fl}$	U(3.00, 6.00)	4.44 (3.12-5.93)	Assumption
Female, high:intermediate risk	$rc_{fh:fi}$	U(10.0, 25.0)	16.4 (10.4-24.5)	[10–23]
25-49:15-24 y	$rc_{o:y}$	U(0.85, 1.00)	0.92 (0.85-0.99)	[4–7]
Pregnant:non-pregnant	$rc_{p:n}$	U(0.85, 1.00)	0.92 (0.86-1.00)	[4–7]
Number of sex acts per person (year^{-1})	n	U(52.0, 104.0)	79.6 (54.6-102.4)	Assumption
Probability of condom use per sex act				
All sex, low risk, 15-24 y, non-pregnant	χ_{slyn}	U(0.25, 0.35)	0.31 (0.26-0.35)	[4–7]
Relative probability of condom use per sex act				
Intermediate:low risk	$r\chi_{i:l}$	U(2.30, 2.50)	2.40 (2.31-2.50)	[4–8]
High:intermediate risk	$r\chi_{h:i}$	U(1.00, 1.15)	1.08 (1.00-1.15)	[11–15,17,19,20–24]
25-49:15-24 y	$r\chi_{o:y}$	U(0.85, 1.00)	0.94 (0.86-1.00)	[4–7]
Pregnant:non-pregnant, low risk	$r\chi_{lp:ln}$	U(0.10, 0.30)	0.20 (0.11-0.29)	[4–7]
Pregnant:non-pregnant, intermediate risk	$r\chi_{ip:in}$	U(0.10, 0.30)	0.20 (0.10-0.30)	[4–7]
Pregnant:non-pregnant, high risk	$r\chi_{hp:hn}$	U(0.85, 1.00)	0.93 (0.86-0.99)	Assumption
Efficacy of condoms per sex act	e	U(0.90, 1.00)	0.95 (0.90-1.00)	[25–28]

Continued...

Description	Symbol	Prior Distribution	Posterior, Median (UI)	Source
**Diagnosis and treatment**				
Probability symptomatic individuals access care				
Male, low risk, 15-24 y, non-pregnant	γ_{mlyn}	U(0.65, 0.75)	0.70 (0.65-0.75)	[4–7]
Relative probability symptomatics access care				
Female:male	$r\gamma_{f:m}$	U(0.70, 0.90)	0.81 (0.70-0.90)	[4–7]
Intermediate:low risk	$r\gamma_{i:l}$	U(1.00, 1.10)	1.05 (1.00-1.10)	Assumption
High:intermediate risk	$r\gamma_{h:i}$	U(1.00, 1.10)	1.05 (1.00-1.10)	[21–23, 29, 30, 5, 6]
25-49 y:15-24 y	$r\gamma_{o:y}$	U(1.00, 1.10)	1.05 (1.00-1.10)	[4–7]
Pregnant:non-pregnant	$r\gamma_{p:n}$	U(1.00, 1.10)	1.05 (1.00-1.10)	[4–7]
Rate of treatment for symptomatic males (year^{-1})	τ^{y}_{m}	U(20.0, 40.0)	29.4 (20.6-39.4)	[31–36, 37–41]
Relative rate of treatment among symptomatics				
Female:male	$r\tau^{y}_{f:m}$	U(0.50, 1.00)	0.76 (0.52-0.98)	[37–41]
Probability of POCT diagnosis among symptomatic	δ_{srag}	Fixed	Scenario specific	Text 1.1.4
Probability of primary healthcare access among all risk, 15-24 y, non-pregnant				
Male	h_{mryn}	Fixed	0.70	[42–46]
Female	h_{fryn}	Fixed	0.75	[42–46]
Relative probability of primary healthcare access among all sex and risk				
25-49 y:15-24 y	$rh_{o:y}$	Fixed	1.05	[46]
Pregnant:non-pregnant	$rh_{p:n}$	Fixed	1.20	[4–7, 47, 48]
Rate of screening with POCT (year^{-1})	τ^{h}_{srag}	Fixed	Scenario specific	Text 1.1.4
Sensitivity of syndromic management				
Male	ζ_m	U(0.80, 0.90)	0.85 (0.80-0.90)	[49]
Female	ζ_f	U(0.40, 0.50)	0.45 (0.40-0.50)	[50, 51]
Sensitivity of POCT				
Male	η_m	Fixed	0.95	[52]
Female	η_f	Fixed	0.95	[52]
**Natural history**				
Probability of transmission per sex act				
Female-to-male	κ_m	U(0.15, 0.30)	0.23 (0.15-0.30)	[36, 53–57]
Male-to-female	κ_f	U(0.10, 0.40)	0.19 (0.10-0.37)	[33, 36, 55–57]
Probability infection is symptomatic				
Male	ϕ_m	U(0.45, 0.90)	0.60 (0.46-0.85)	[35, 36, 55, 56, 58, 59]
Female	ϕ_f	U(0.20, 0.45)	0.35 (0.22-0.44)	[35, 36, 55, 56, 58–60]
Rate of natural recovery (year^{-1})	π	U(1.80, 2.80)	2.31 (1.84-2.76)	[35, 36, 56, 61–63]
Rate of recovery with treatment (year^{-1})	σ	U(26.0, 52.0)	39.0 (26.6-51.5)	[64]
Probability syndrome is caused by gonorrhoea				
Male (urethral discharge)	p_m	U(0.65, 0.8)	0.73 (0.65-0.80)	[65]
Female (vaginal discharge)	p_f	U(0.10, 0.20)	0.14 (0.10-0.20)	[65]

Row shading indicates fixed (light blue) or estimated (white) parameters. CFSW: clients of female sex workers, FSW: female sex workers, UI: Uncertainty interval, U: Uniform distribution (min, max), y: years.

1.3 Transmission model calibration

Calibration was performed using a single-round rejection-based approach to accommodate broadly defined calibration targets arising from sparse prevalence data, particularly for higher-risk and male populations. Our primary objective was to identify an ensemble of plausible parameter sets and to assess the robustness of intervention scenario comparisons and prioritisation across this range.

1.3.1 Calibration outcomes

Calibration outcomes included: (i) gonorrhoea prevalence among sexually active females in the general population, (ii) gonorrhoea prevalence among FSW, (iii) male-to-female ratio for gonorrhoea prevalence, and (iv) male-to-female ratio for all-cause symptomatic case rates (Table S3). Due to limited studies, gonorrhoea prevalence among males was not used as a direct calibration outcome. Instead, we used ratios for male-to-female gonorrhoea prevalence and male-to-female all-cause symptomatic case rate. Calibration target ranges for the first three outcomes were derived from meta-regression estimates for 2020, and applied to equilibrium outcomes in the model baseline scenario in 2025.

Table S3: Calibration outcomes

Variable	Target	Estimate (95%CI) informing target	Source for estimate
**Gonorrhoea prevalence**			
Female, overall	U(1.0%, 3.5%)	2.2% (1.3 - 3.5%)	Meta-regression estimate in 2020 (Figure S2)
Female sex workers	U(2.0%, 6.5%)	3.7% (2.1 - 6.4%)	Meta-regression estimate in 2020 (Figure S2)
**Male-to-female ratios**			
Prevalence	U(0.60, 1.00)	0.81 (0.61 - 1.09)	Meta-regression estimate in 2020[66]
Symptomatic case rate	U(0.35, 0.60)	0.46 (0.36 - 0.58)	Analysis of Kenya DHS[4–6]

Symptomatic case rate: annual number of symptomatic urethral or vaginal discharge cases due to any cause, divided by the sexually active population size.

1.3.2 Prevalence estimates

Gonorrhoea prevalence was estimated through systematic data collation and meta-regression. For the general population, we extracted prevalence observations (n = 24) from Kenyan studies between 2000 and 2024, with diagnostic test performance adjustment, from our previous systematic review.[66] These studies primarily involved sexually active populations. For FSW, we conducted an additional PubMed search using the same inclusion criteria, with an added search domain for sex work (i.e. “sex work”, “sex worker”, “prostitute”, “commercial sex”). After de-duplication of identified articles, prevalence observations (n = 12) were extracted and adjusted for diagnostic test performance using the same approach as in our prior analysis (Table S4).[66]

A generalised linear mixed-effects meta-regression model was then fit to the collated data. Fixed effects included year (midpoint of data collection), population group (general population, FSW), sex (female, male), age group (younger adults 12–25 years, all adults $\geq$ 12 years), and HIV status (HIV negative, non-stratified status). Interaction terms between year and sex, and year and population group, were not included due to limited observations. A study-level random intercept accounted for between-study heterogeneity. Model predictions were used to estimate prevalence for adults in the general and FSW populations, without stratifying by HIV status (Figure S2). Predictions were not stratified by age group, due to limited observations among younger adults (n = 6 general population, n = 0 FSW).

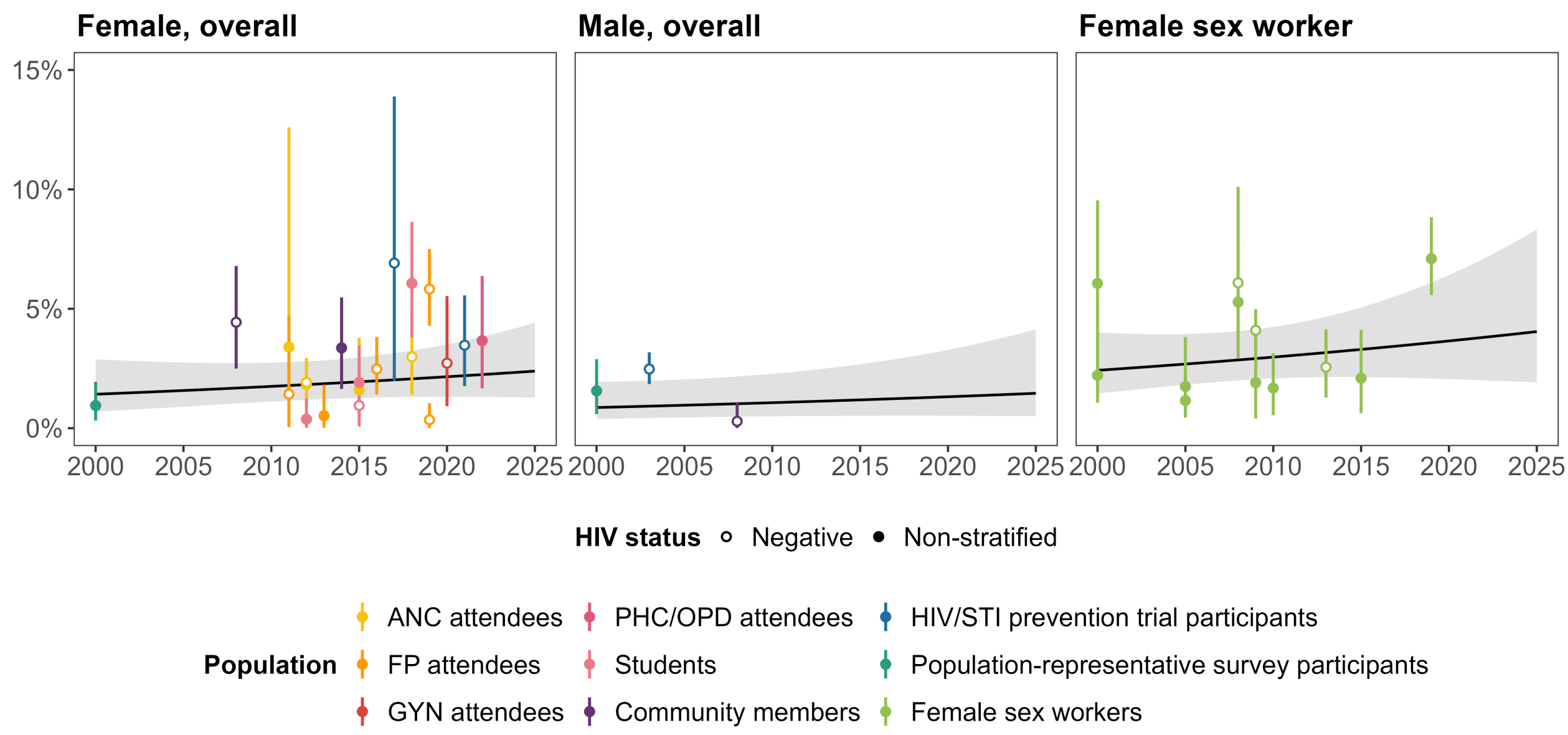

Figure S2: Estimated prevalence of gonorrhoea among the general population and female sex workers in Kenya, 2000-2025

Gonorrhoea prevalence among male and female general populations and FSW between 2000-2025. Lines and shading depict mean estimates with 95% CIs. Points represent study observations. ANC: antenatal care, FP: family planning, GYN: gyneacology, PHC/OPD: primary healthcare or outpatient department.

Table S4: Study observations of gonorrhoea prevalence in Kenya

Sex	Population	Year	Setting	Age	HIV status	Samp	Test	Sens (%)	Spec (%)	N	Prevalence Raw (%)	Prevalence Adjusted (%, 95%CI)	Reference
**Population category: General**													
F	Population-representative survey participants	2000	Mombasa	15 to 49	Non-stratified	UR	NAAT	91.6	100.0	571	0.7	0.9 (0.3-1.9)	Hawken 2002[67]
F	Community members	2008	Kisumu	18 to 34	Negative	GE	NAAT	93.3	99.2	424	4.7	4.4 (2.5-6.8)	Otieno 2015[68]
F	ANC attendees	2011	Mombasa	18 to 35	Non-stratified	GE	NAAT	93.3	99.2	30	0.0	3.4 (0.1-12.6)	Jespers 2014[69]
F	FP attendees	2011	Mombasa	18 to 35	Negative	GE	NAAT	93.3	99.2	110	0.9	1.4 (0.0-4.7)	Jespers 2014[69]
F	ANC attendees	2012	Western Region	14+	Negative	GE	NAAT	93.3	99.2	1276	2.5	1.9 (1.1-2.9)	Kinuthia 2015[70]
F	Students	2012	Western Region	14 to 17	Non-stratified	GE	NAAT	93.3	99.2	511	0.6	0.4 (0.0-1.2)	Kerubo 2016[71]
F	FP attendees	2013	Nairobi	20 to 49	Non-stratified	GE	Culture	75.7	100.0	249	0.0	0.5 (0.0-1.8)	Maina 2016[72]
F	Community members	2014	Kisumu	18 to 34	Non-stratified	GE	NAAT	93.3	99.2	457	3.7	3.4 (1.6-5.5)	Oliver 2018[73]
F	ANC attendees	2015	Kilifi	18 to 45	Non-stratified	UR	NAAT	91.6	100.0	202	1.0	1.6 (0.3-3.8)	Masha 2017[74]
F	Students	2015	Thika	16 to 20	Negative	GE	NAAT	93.3	99.2	373	1.3	1.0 (0.1-2.5)	Yuh 2020[75]
F	FP attendees	2016	Kisumu	16 to 35	Negative	GE	NAAT	93.3	99.2	901	3.0	2.5 (1.4-3.8)	Deese 2021[76]
F	Prevention trial participants	2017	Kisumu	18 to 50	Negative	GE/ UR	NAAT	91.6	99.2	82	6.1	6.9 (2.0-13.9)	Mgodi 2021[77]
F	ANC attendees	2018	NR	NR	Negative	GE	NAAT	93.3	99.2	474	3.4	3.0 (1.4-4.9)	Madanitsa 2023[78]
F	Students	2018	Siaya	14 to 22	Non-stratified	GE	NAAT	93.3	99.2	436	6.2	6.1 (3.8-8.6)	Mehta 2023[79]
F	FP attendees	2019	Nairobi, Mombasa	18 to 45	Negative	GE	NAAT	93.3	99.2	691	0.7	0.4 (0.0-1.0)	Lokken 2022[80]
F	FP attendees	2019	Kisumu	16 to 25	Negative	GE	NAAT	93.3	99.2	1000	6.1	5.8 (4.3-7.5)	Celum 2022[81]
F	GYN attendees	2020	Thika, Kisumu	15 to 30	Negative	UR	NAAT	91.6	100.0	198	2.0	2.7 (0.9-5.5)	Heffron 2021[82]

Continued...

Sex	Population	Year	Setting	Age	HIV status	Samp	Test	Sens (%)	Spec (%)	N	Prevalence Raw (%)	Prevalence Adjusted (%, 95%CI)	Reference
F	Prevention trial participants	2021	Kisumu	18 to 30	Negative	GE	NAAT	93.3	99.2	448	3.8	3.5 (1.8-5.6)	Oware 2023[83]
F	OPD attendees	2022	Kitui	15 to 44	Non-stratified	GE	Culture	75.7	100.0	322	2.5	3.7 (1.7-6.4)	Mbuvi 2024[84]
M	Population-representative survey participants	2000	Mombasa	15 to 49	Non-stratified	UR	NAAT	80.9	99.9	583	1.2	1.6 (0.6-2.9)	Hawken 2002[67]
M	Prevention trial participants	2003	Kisumu	18 to 24	Negative	UR	NAAT	80.9	99.9	2754	2.1	2.5 (1.8-3.2)	Bailey 2007[85]
M	Community members	2008	Kisumu	18 to 34	Negative	UR	NAAT	80.9	99.9	422	0.0	0.3 (0.0-1.1)	Otieno 2015[68]
**Population category: Female sex workers**													
F	Female sex workers	2000	Nairobi	18 to 35	Non-stratified	GE	NAAT	93.3	99.2	295	6.1	6.1 (3.4-9.3)	Cohen 2007[86]
F	Female sex workers	2000	Mombasa	15 to 65	Non-stratified	UR	NAAT	91.6	100.0	493	1.8	2.2 (1.1-3.8)	Hawken 2002[12]
F	Female sex workers	2005	Kisumu	NR	Non-stratified	UR	NAAT	91.6	100.0	250	1.2	1.7 (0.4-3.8)	Kwena 2010[87]
F	Female sex workers	2005	Mombasa	16+	Non-stratified	GE	Culture	75.7	100.0	677	0.7	1.2 (0.5-2.3)	Chersich 2007[88]
F	Female sex workers	2008	Nairobi	18 to 55	Negative	GE	NAAT	93.3	99.2	200	6.0	6.1 (2.8-10.3)	Priddy 2011[14]
F	Female sex workers	2008	Kisumu	NR	Non-stratified	GE	NAAT	93.3	99.2	474	5.5	5.3 (3.2-7.8)	Vandenhoudt 2013[15]
F	Female sex workers	2009	Nairobi	18 to 49	Non-stratified	GE	NAAT	93.3	99.2	348	2.3	1.9 (0.4-3.9)	Gomih-Alakija 2014[16]
F	Female sex workers	2009	Nairobi	NR	Negative	GE	Culture	75.7	100.0	2931	3.1	4.1 (3.3-5.0)	Preston 2013[89]
F	Female sex workers	2010	Nairobi	18+	Non-stratified	GE	NAAT	93.3	99.2	596	2.2	1.7 (0.5-3.1)	Musyoki 2015[17]
F	Female sex workers	2013	Mombasa	NR	Negative	GE	NAAT	93.3	99.2	660	3.0	2.6 (1.3-4.1)	Willcox 2021[19]
F	Female sex workers	2015	Mombasa	19 to 66	Non-stratified	GE	NAAT	93.3	99.2	399	2.5	2.1 (0.6-4.0)	Grabert 2022[90]
F	Female sex workers	2019	Nairobi	18 to 45	Non-stratified	UR	NAAT	91.6	100.0	1042	6.4	7.1 (5.6-8.8)	Beksinska 2021[20]

Study year is midpoint between start and end of data collection period. Sensitivity and specificity of diagnostic tests collated per approach outlined by Michalow *et al.* (2025).[66] ANC: antenatal care, FP: family planning, F: female, GE: genital fluid, GYN: gynaecology clinic M: male, OPD: outpatient department, samp: sample, sens: sensitivity, spec: specificity, UR: urine, NR: not reported.

1.4 Quality-adjusted life years lost

1.4.1 Probability trees and parametrisation

Probability tree structures for sequelae in men, non-pregnant women, and pregnant women are shown in Figure S3. Frameworks for men and non-pregnant women were from Li *et al*,[91] while a new structure was developed to capture pregnancy outcomes.

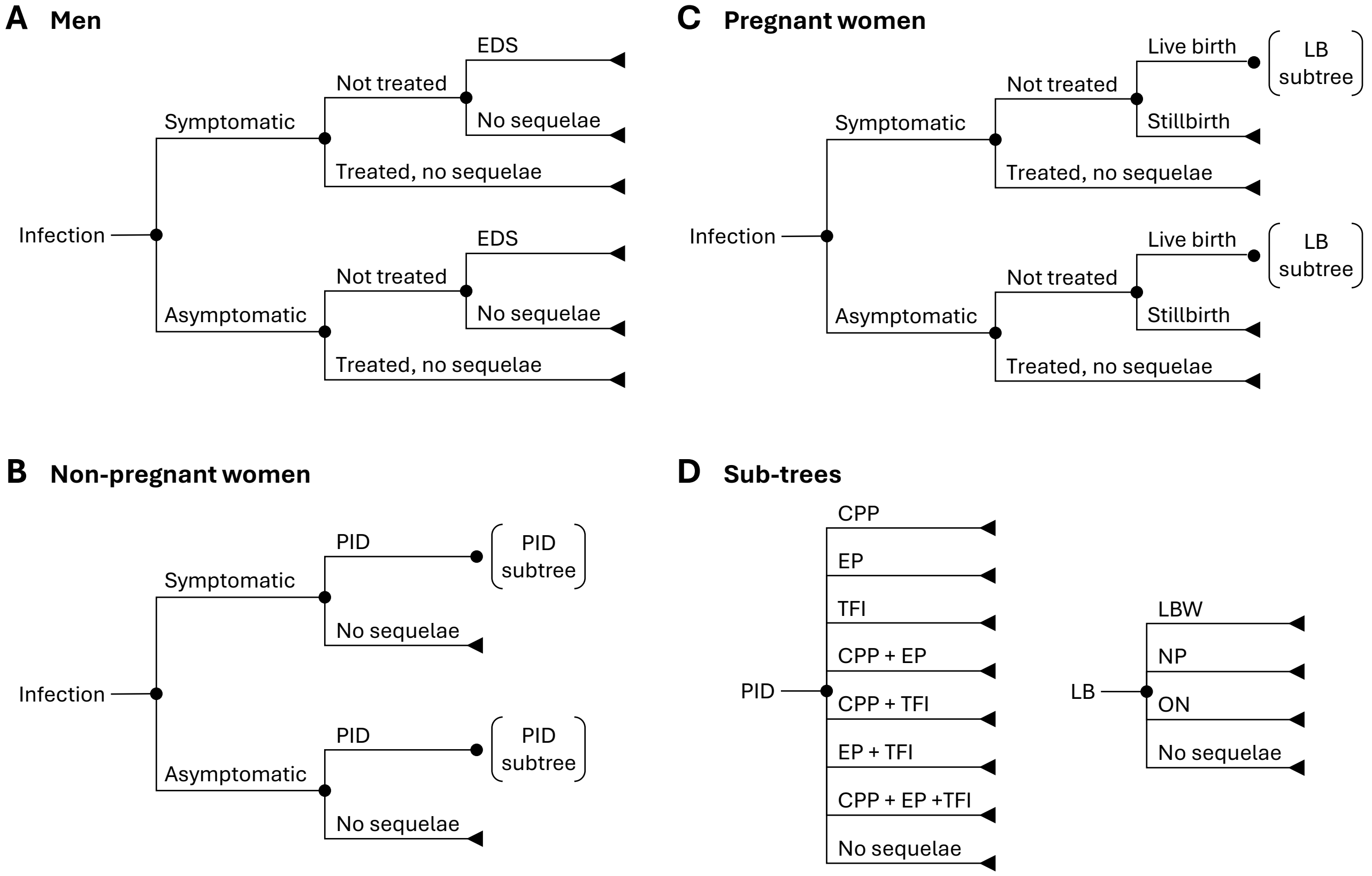

Figure S3: Probability trees for gonorrhoea sequelae

Probability trees for sequelae of gonorrhoea among (A) men, (B) non-pregnant women, and (C) pregnant women, with complications of PID and LB shown as separate subtrees (D). CPP: chronic pelvic pain, EDS: epididymo-orchitis, EP: ectopic pregnancy, LB: live birth, LBW: low birth weight, NP: neonatal pneumonia, ON: ophthalmia neonatorum, PID: pelvic inflammatory disease, TFI: tubal factor infertility. Trees and subtrees for men and non-pregnant women adapted from Li *et al.* 2023.[91]

Health losses were quantified as quality-adjusted life years (QALYs), where one QALY equals one year lived in perfect health. Each health state was assigned a utility value between 0 (death) and 1 (perfect health), where a utility decrement (1 - utility) represents the fraction of a healthy year lost due to that outcome. To calculate QALY losses at the population level, we used posterior estimates of gonorrhoea incidence and duration, distinguishing between episodes that were symptomatic or asymptomatic, and treated or untreated. Untreated episodes included infections receiving no treatment as well as infections receiving inappropriate treatment that did not clear gonorrhoea. We did not assign a separate utility decrement to inappropriate treatment. For acute infection episodes (urethritis or

symptomatic cervicitis), we multiplied person-years of infection for each subgroup by the corresponding utility decrement to capture shorter-term reductions in quality of life. For longer-term sequelae, we multiplied the number of incident infections by the probability that each sequela would result from an infection episode, the duration of the sequela, and its associated utility decrement.

Parameter values for probabilities, durations, and utilities were obtained from published literature (Table S5). Sequelae probabilities were mainly derived from longitudinal studies of chlamydia, due to sparse direct evidence for gonorrhoea.[91,92] Values for utility weights and their associated durations were from the Global Burden of Disease study,[93,94] the Institute of Medicine study,[95] and other published literature, with DALY disability weights converted to health-state utility values as 1 minus the disability weight, consistent with previous studies.[91] CPP and TFI were classified as chronic sequelae with onset 5 years post-infection and duration of 10 years.[91,96] Stillbirth losses were values as equivalent to neonatal deaths (utility weight of 0 over life expectancy at birth), due to limited consensus on alternative quantification approaches.[97] Mothers with stillbirth were assigned a utility reduction for 10 years to reflect long-term psychological impacts.[98] Utility decrements were also included for mothers of infants with LBW, NP, or ON.[99]

Table S5: Parameters for the probability, duration, and utility of gonorrhoea sequelae

Sequelae	Probability per infection episode	Duration (years)	Utility weight
**Men**			
Urethritis, given symptomatic infection	1.000 [91]	Simulated*	0.961 [93,95]
Urethritis, given asymptomatic infection	0 [91]	Simulated*	1.000 [93]
EDS, given untreated infection	0.043 [91]†	0.019 [95]	0.665 [93,95]
**Non-pregnant women**			
Symptomatic infection	Simulated*	Simulated*	0.994 [93]
Asymptomatic infection	Simulated*	Simulated*	1.000 [93]
PID	Simulated with annual rate ‡	0.028 [95]	0.756 [93,95]
CPP, given PID	0.261 [91]†	10.00 [91,96]	0.759 [93,95]
TFI, given PID	0.168 [91]†	10.00 [91,96]	0.905 [93,95]
EP, given PID	0.070 [91]†	0.078 [95]	0.731 [93,95]
**Pregnant women**			
Symptomatic infection	Simulated*	Simulated*	0.994 [93]
Asymptomatic infection	Simulated*	Simulated*	1.000 [93]
Stillbirth, given untreated infection	0.047 ‖	Neonatal: 64.00 [3]<br>Maternal: 10.00 [98]	Neonatal: 0<br>Maternal: 0.862 [98]
LBW, given LB with untreated infection	0.176 ‖	Neonatal: 64.00 [3]<br>Maternal: 10.00 §	Neonatal: 0.894 [94]<br>Maternal: 0.999 §
NP, given LB with untreated infection	0.160 [100]†	0.167 [95]	Neonatal: 0.790 [95]<br>Maternal: 0.999 §
ON, given LB with untreated infection	0.180 [100]†	0.500 [95]	Neonatal: 0.970 [95]<br>Maternal: 0.999 §

Utilities with two sources were averaged. *Probability and duration of symptomatic and asymptomatic gonorrhoea varied per posterior parameter combination. †Probabilities of sequelae approximated from studies of chlamydia, due to limited gonorrhoea-specific data.[91] ‡Probability of PID calculated using mean annual progression rate of 0.15 per year[101] and posterior estimates for duration of infection.[91] ‖Probabilities of stillbirth and LBW among pregnant women with untreated gonorrhoea estimated by combining odds ratios from meta-analyses with background prevalence in the general population of pregnant women.[102–104] §Assumption.[99] CPP: chronic pelvic pain, EDS: epididymitis, EP: ectopic pregnancy, LB: live birth, LBW: low birth weight, NP: neonatal pneumonia, ON: ophthalmia neonatorum, PID: pelvic inflammatory disease, TFI: tubal factor infertility.

1.4.2 Equations for individual and combined sequelae

QALYs lost were calculated separately for individual and combined sequelae. In the notation used below, subscripts s and ℓ refer to shorter- and longer-term sequelae, respectively. For individual sequelae (whether short- or long-term), QALYs lost (Q) were calculated as the product of the number of incident gonorrhoea cases (x), the probability of developing the sequela (p), the duration of utility loss (d), and the decrement in health utility ($1-u$):

$$Q = x\,p\,d\,(1-u) \tag{1.33}$$

For combined sequelae following PID, QALYs lost were calculated in two parts, consistent with the approach used by Li *et al.*[91] covering: (i) the overlapping period, during which both sequelae occurred simultaneously, corresponding to the duration of the shorter-term sequela (d_s); and (ii) the extended period, during which only the longer-term sequela persisted, defined as the remaining duration of the longer-term sequela ($d_\ell - d_s$ where $d_\ell > d_s$). During the overlapping period, the joint utility was calculated as the product of the individual utility weights ($u_s\,u_\ell$). During the extended period, only the utility of the longer-term sequela (u_ℓ) was applied. The total QALY loss among individuals with combined sequelae was:

$$Q_{s\ell} = x\ p_s\ p_\ell\ [(1-u_s\,u_\ell)\,d_s + (1-u_\ell)(d_\ell - d_s)] \tag{1.34}$$

Here, x denoted the estimated number of PID cases, p_s and p_ℓ were the probabilities of developing each sequela, u_s and u_ℓ were the respective utility weights, and d_s and d_ℓ were the corresponding durations.

For sequelae extending beyond the year of infection, durations were calculated using a life table approach and were discounted to the year of infection.[91]

1.4.3 Adverse birth outcomes

To quantify QALYs lost due to stillbirth and LBW, estimates were required for the probability of each outcome among pregnant women with untreated gonorrhoea. However, direct estimates of these probabilities are rarely available. Most studies report associations using odds ratios (ORs), which quantify the relative odds of an outcome in exposed versus unexposed groups, i.e., among women with untreated gonorrhoea compared to those without gonorrhoea or with treated infection. ORs alone cannot estimate absolute risks, since they do not account for underlying prevalence of the outcome in the general population. Therefore, we used the approach described by Nyemba *et al.*[105] to convert ORs into absolute probabilities suitable for QALY calculations, by linking relative and absolute measures through algebraic relationships.

The approach involved expressing the outcome probability among exposed individuals (p_1) and unexposed individuals (p_0) using both the OR and risk ratio (RR):

$$\text{OR} = \tfrac{p_1}{1-p_1} / \tfrac{p_0}{1-p_0} \quad (1.35)$$

$$\text{RR} = \frac{p_1}{p_0} \quad (1.36)$$

To express RR in terms of OR, Equation 1.35 was substituted into 1.36:

$$\text{RR} = \frac{\text{OR}}{1 - p_0(1 - \text{OR})} \quad (1.37)$$

The background prevalence of the adverse outcome in the general pregnant population (u) was calculated using the median equilibrium prevalence of untreated gonorrhoea at baseline (g):

$$u = g \cdot p_1 + (1 - g) \cdot p_0 \quad (1.38)$$

Substituting Equation 1.37 and solving for p_0 yielded:

$$p_0 = \frac{u}{1 + g \cdot (\frac{\text{OR}}{1-p_0(1-\text{OR})} - 1)} \quad (1.39)$$

Equation 1.39 was rearranged into a quadratic Equation:

$$a \cdot p_0^2 + b \cdot p_0 + c = 0 \quad (1.40)$$

Where the coefficients were defined as:

$a = (1 - g)(\text{OR} - 1)$

$b = 1 - (\text{OR} - 1)(u - g)$

$c = -u$

The value for p_0 was obtained using the quadratic formula: $p_0 = \frac{-b \pm \sqrt{b^2-4ac}}{2a}$

The value for p_1 was then calculated using Equation 1.35.

The odds ratios, background prevalence, and resulting probability estimates (p_1) are in Table S6.

Table S6: Probability of adverse outcomes in pregnant women with untreated gonorrhoea.

Parameter	Stillbirth	Low birth weight
Odds ratio (effect of gonorrhoea on outcome)	2.16 (1.25-3.46)[104]	1.66 (1.12 - 2.48)[104]
Background prevalence of outcome in general pregnant population	0.023[102]	0.115[103]
Estimated probability of outcome in pregnant women with untreated gonorrhoea	0.047	0.176
Estimated probability of outcome in pregnant women with treated gonorrhoea or no gonorrhoea	0.023	0.114

1.4.4 Duration and discounting

Long-term durations were assigned to chronic sequelae of PID (CPP and TFI) in non-pregnant women and adverse birth outcomes (stillbirth and LBW) in pregnant women and their infants. For chronic sequelae, a 10-year duration was assumed, representing a conservative intermediate between the 5-year, 10-year, and lifetime durations considered by Li *et al.*[91] Stillbirth and LBW losses were valued using life expectancy at birth (65 years).[3, 94, 106–108] For mothers, stillbirth-associated losses were valued over 10 years based on maternal perinatal mortality surveys,[98] and LBW-associated maternal impacts were assumed to have the same 10-year duration.

Years lived with long-term sequelae were estimated assuming infections and sequelae onset at age group midpoints: age 20 for the 15-24 year group and age 37 for the 25-49 years group. For chronic sequelae, onset was modelled with a 5 year lag after infection, with health losses occurring from ages 25-35 years for the younger group and 42-52 years for the older group. Years lived were calculated using a life table approach with discounting applied from the year of infection.[91] Life tables were constructed using five-year age intervals for the conditional probabilities of all-cause mortality and survival from UN WPP projections.[3] Deaths were assumed to occur at the midpoint of each age interval. Age weights were not applied, per GBD and WHO conventions valuing health losses equally across age.[109, 110]

For each interval, the undiscounted years lived (YL_u) were calculated by incorporating the probability of surviving from age at infection to the start of the age interval (p_i), and the probability of death during the interval (p_d).

$$\mathrm{YL}_u = p_i\,[2.5\,p_d + 5\,(1 - p_d)] \tag{1.41}$$

The years lived were then discounted (YL_d) for each interval using a 3% annual discount rate applied from infection onset to the interval midpoint:

$$\mathrm{YL}_d = \sum_{k=0}^{n-1} \frac{1}{(1+r)^{t+k}} \tag{1.42}$$

Where r was the annual discount rate, t was the number of years from infection onset to interval start (including sequelae onset delays), n was the number of years with the sequela, and k indexed each year within the interval.

Undiscounted and discounted years lived were summed across relevant age intervals (Table S7) and used to calculate lifetime QALYs lost due to each sequela.

Table S7: Years lived with health losses due to each sequela

Sequelae	Age group	Age of infection	Duration sequela	Years lived	
				Undiscounted	Discounted
Chronic: CPP and TFI	15-24 years	20	10	9.75	7.28
Chronic: CPP and TFI	25-49 years	37	10	9.23	6.92
Maternal: Stillbirth and LBW	15-24 years	20	10	9.89	8.56
Maternal: Stillbirth and LBW	25-49 years	37	10	9.59	8.46
Stillbirth and LBW	Neonate	0	64	56.5	26.3

2 Supplementary results

2.1 Transmission model calibration

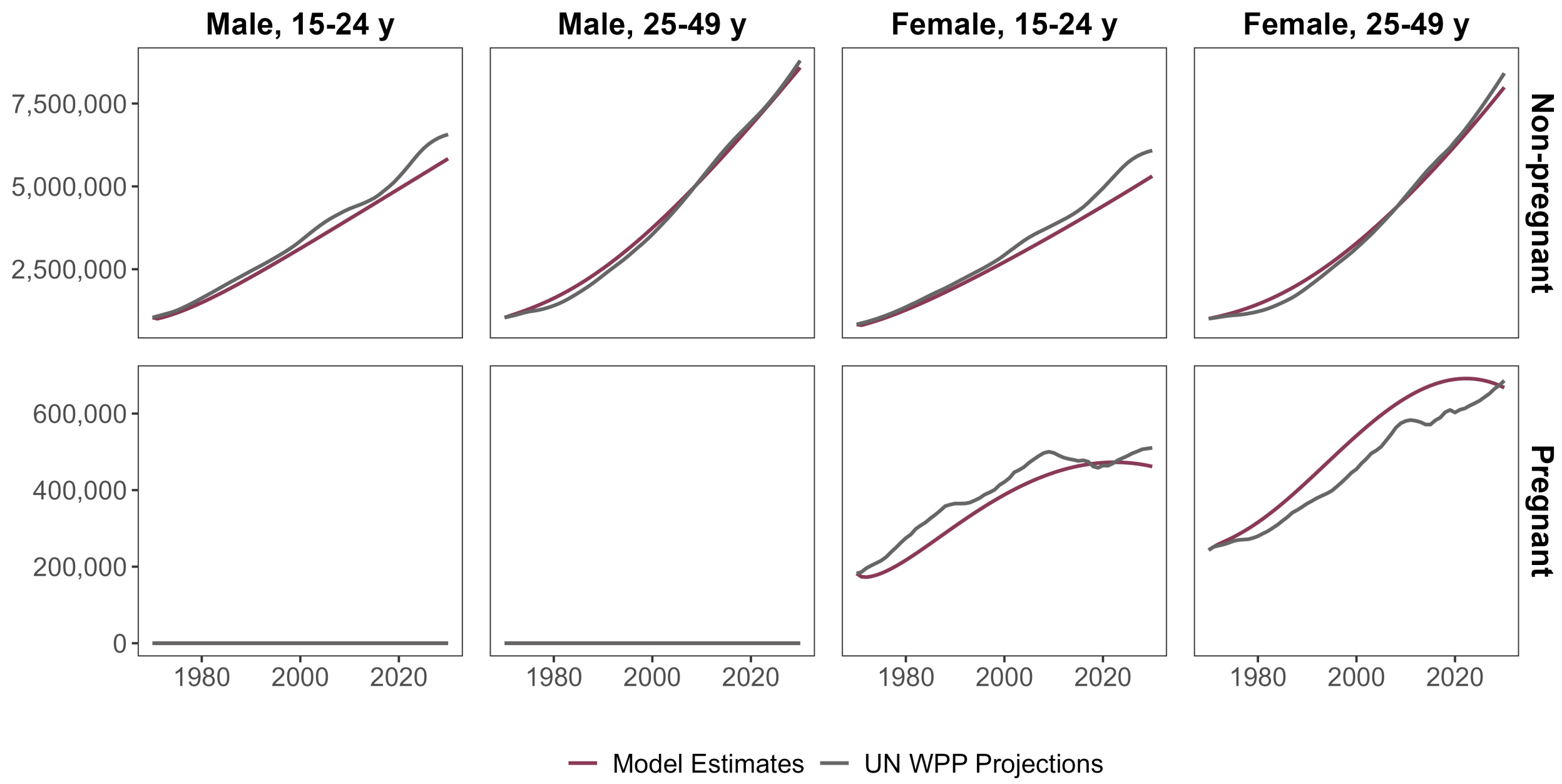

Figure S4: Comparison of model population estimates with UN WPP projections

Deviations between model estimates and UN WPP annual projections resulted from demographic parameters in the model, which were derived from cohort-based calculations and linearly interpolated between 1970 and 2030.

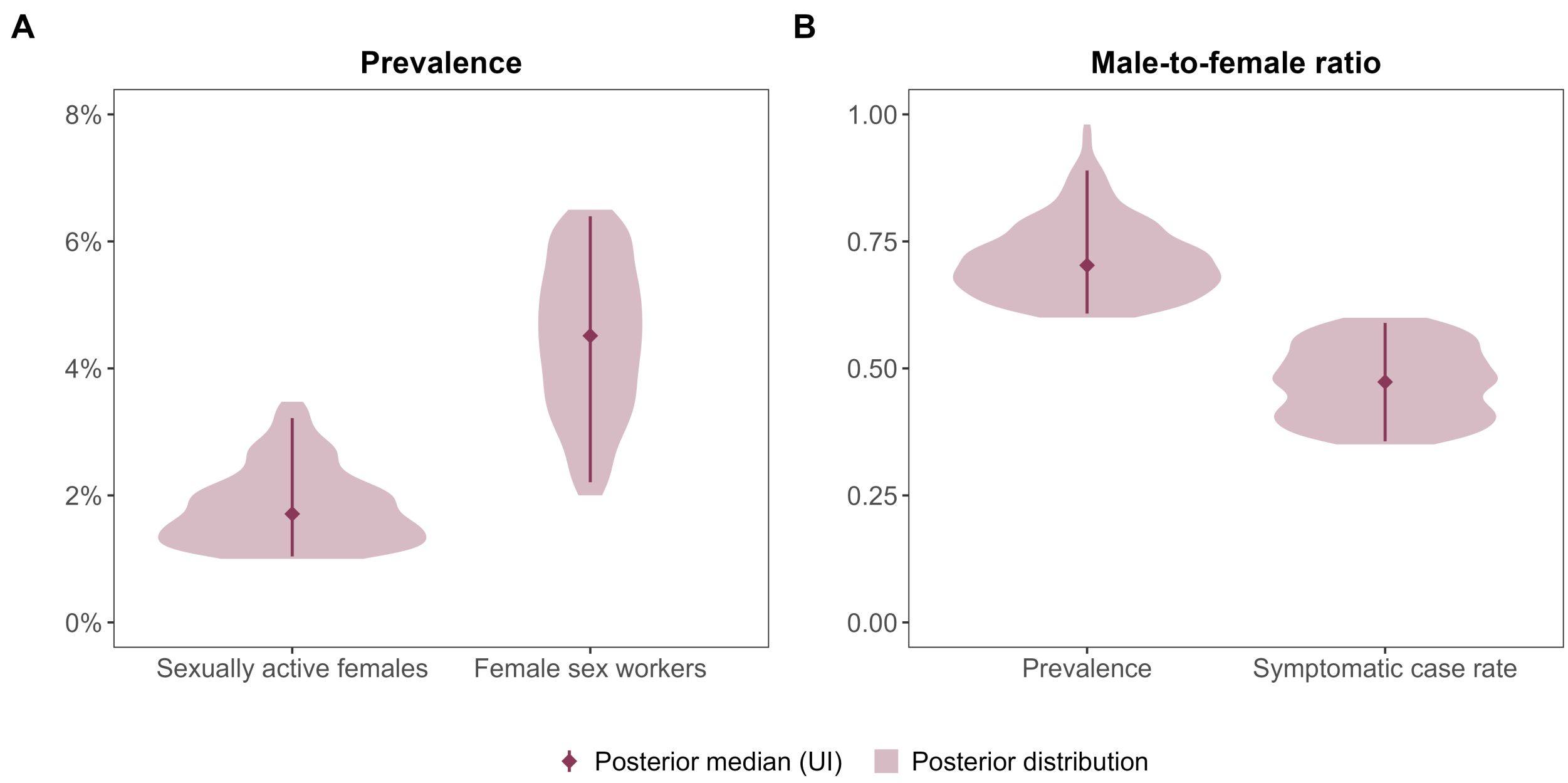

Figure S5: Distribution of baseline model outcomes after calibration

Posterior distributions of baseline model outcomes within calibration target ranges at equilibrium in 2025, for (A) gonorrhoea prevalence among sexually active females and FSW and (B) male-to-female ratios for prevalence and symptomatic case rates. Shaded areas represent the distribution of each outcome across retained posterior samples; points show the median, and vertical lines indicate the 95% uncertainty interval. Calibration target ranges for prevalence and the male-to-female prevalence ratio were derived from meta-regression estimates for 2020 (Figure S2, Table S3).

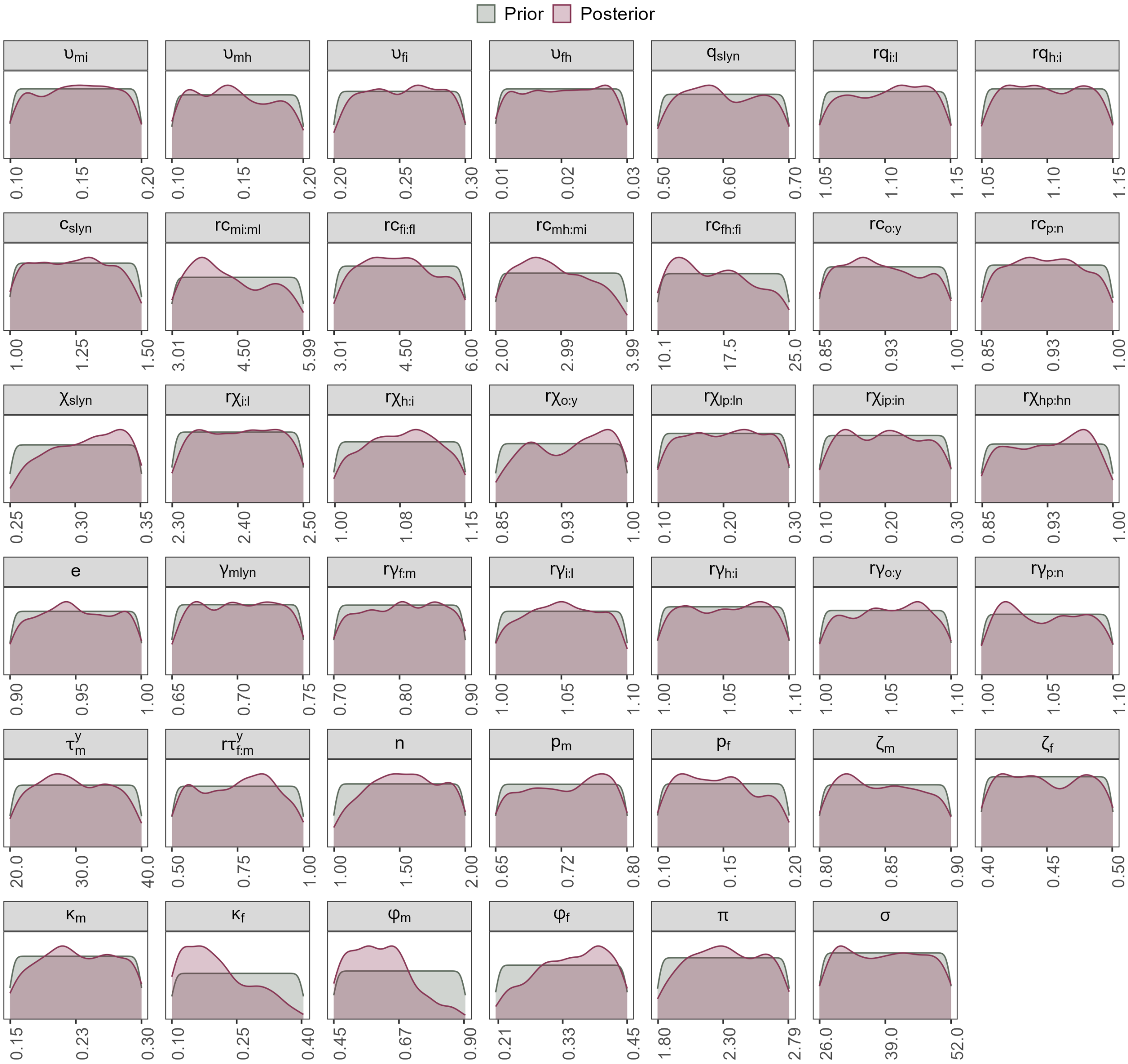

Figure S6: Prior and posterior distributions for calibrated parameters

Distributions for calibrated model parameters, stratified by prior (50 000 initial samples) and posterior (536 retained samples). Parameters are defined in Table S2.

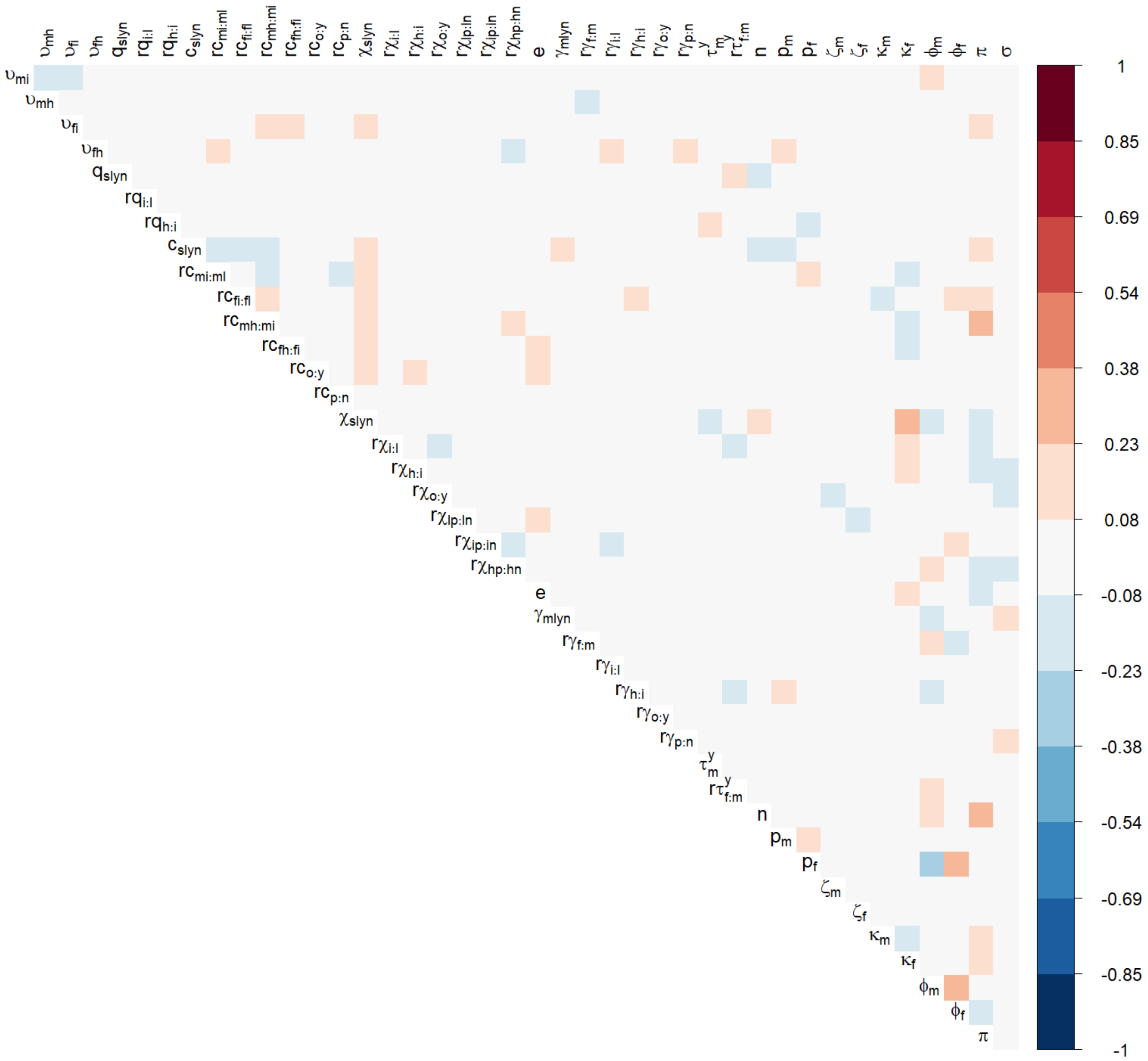

Figure S7: Rank correlations among posterior samples for calibrated model parameters

Parameters are defined in Table S2.

2.2 Baseline outcomes

Table S8: Predicted gonorrhoea burden at baseline, 2025

Variable	CFSW	FSW	Men	Pregnant	AGYW
Population size (millions)	1.91 (1.35 - 2.58)	0.26 (0.14 - 0.39)	13.1 (Fixed)*	1.16 (1.13 - 1.19)	4.85 (4.82 - 4.88)
Prevalence (%)	3.56 (1.99 - 6.74)	4.74 (2.42 - 6.56)	1.28 (0.74 - 2.34)	1.89 (1.14 - 3.59)	1.79 (1.10 - 3.32)
Incident infections per 100 individuals, per priority population	13.1 (6.88 - 24.8)	11.9 (5.67 - 18.6)	4.62 (2.70 - 8.29)	4.97 (2.79 - 9.14)	4.68 (2.76 - 8.52)
QALYs lost per 1000 individuals, per priority population	0.56 (0.27 - 1.19)	22.8 (11.1 - 36.0)	0.21 (0.11 - 0.45)	80.1 (45.1 - 145)	1.98 (1.21 - 3.67)
Proportion total incident infections in priority population (%)	21.4 (13.7 - 29.3)	2.48 (1.00 - 5.55)	50.0 (45.4 - 55.0)	5.37 (4.57 - 6.41)	14.5 (12.6 - 16.5)
Proportion total QALYs lost in priority population (%)	0.84 (0.48 - 1.60)	4.42 (1.67 - 10.4)	2.07 (1.31 - 3.58)	80.6 (76.1 - 83.8)	5.69 (4.53 - 7.20)

Results presented as medians with uncertainty intervals and rounded to three significant figures. *Total population size by sex and age is fixed to match annual UN WPP projections.[3] Variation introduced in population size estimate for AGYW due to calibrated probability of being ever sexually active. AGYW: sexually active adolescent girls and young women; CFSW: clients of female sex workers; FSW: female sex workers.

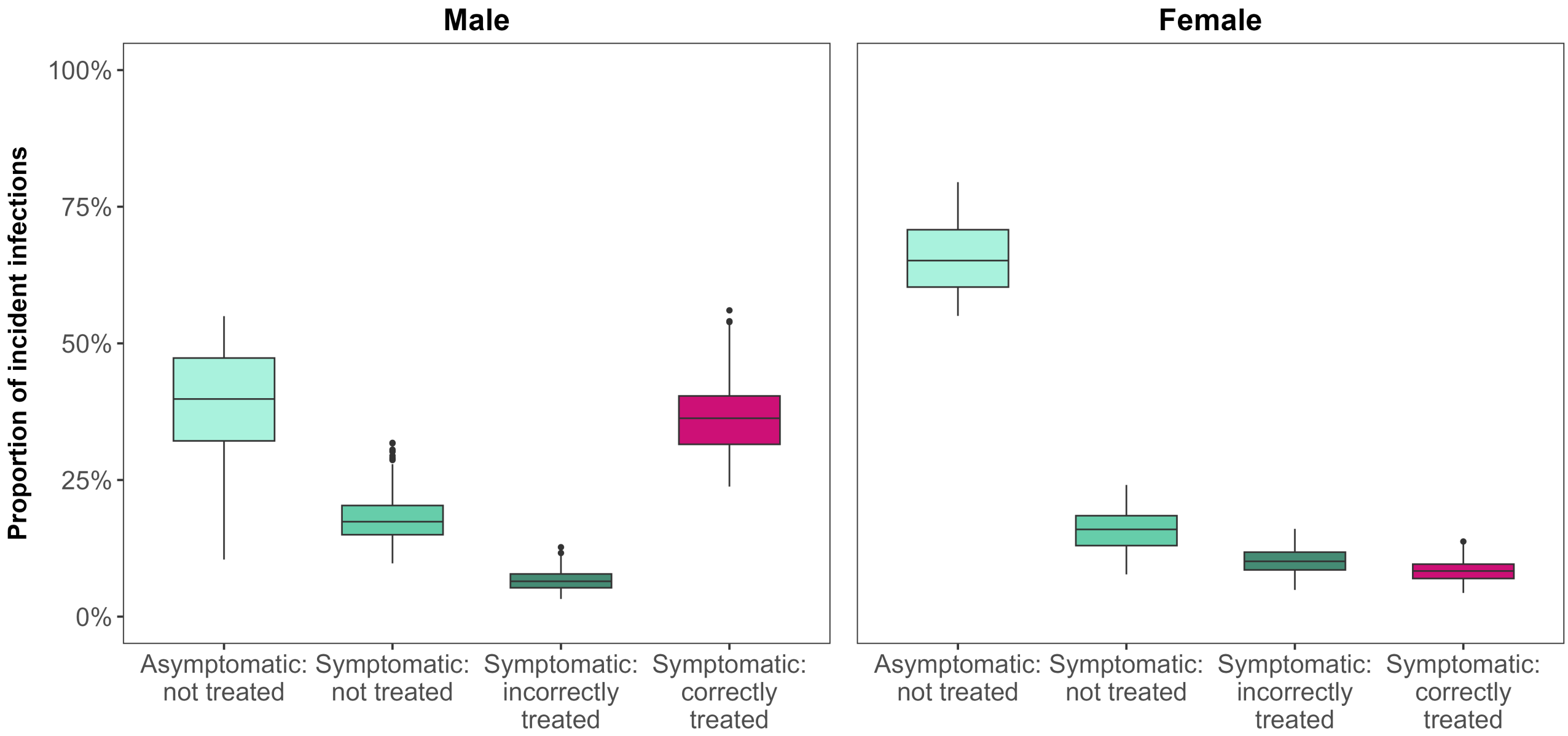

Figure S8: Distribution of incident gonorrhoea infections by symptom and treatment status at baseline, 2025

Proportion of incident gonorrhoea infections in 2025 that were asymptomatic and did not access care, symptomatic and did not access care, symptomatic and accessed care but received incorrect treatment, or symptomatic and accessed care and received correct treatment. Proportions presented as medians with 95% uncertainty intervals.

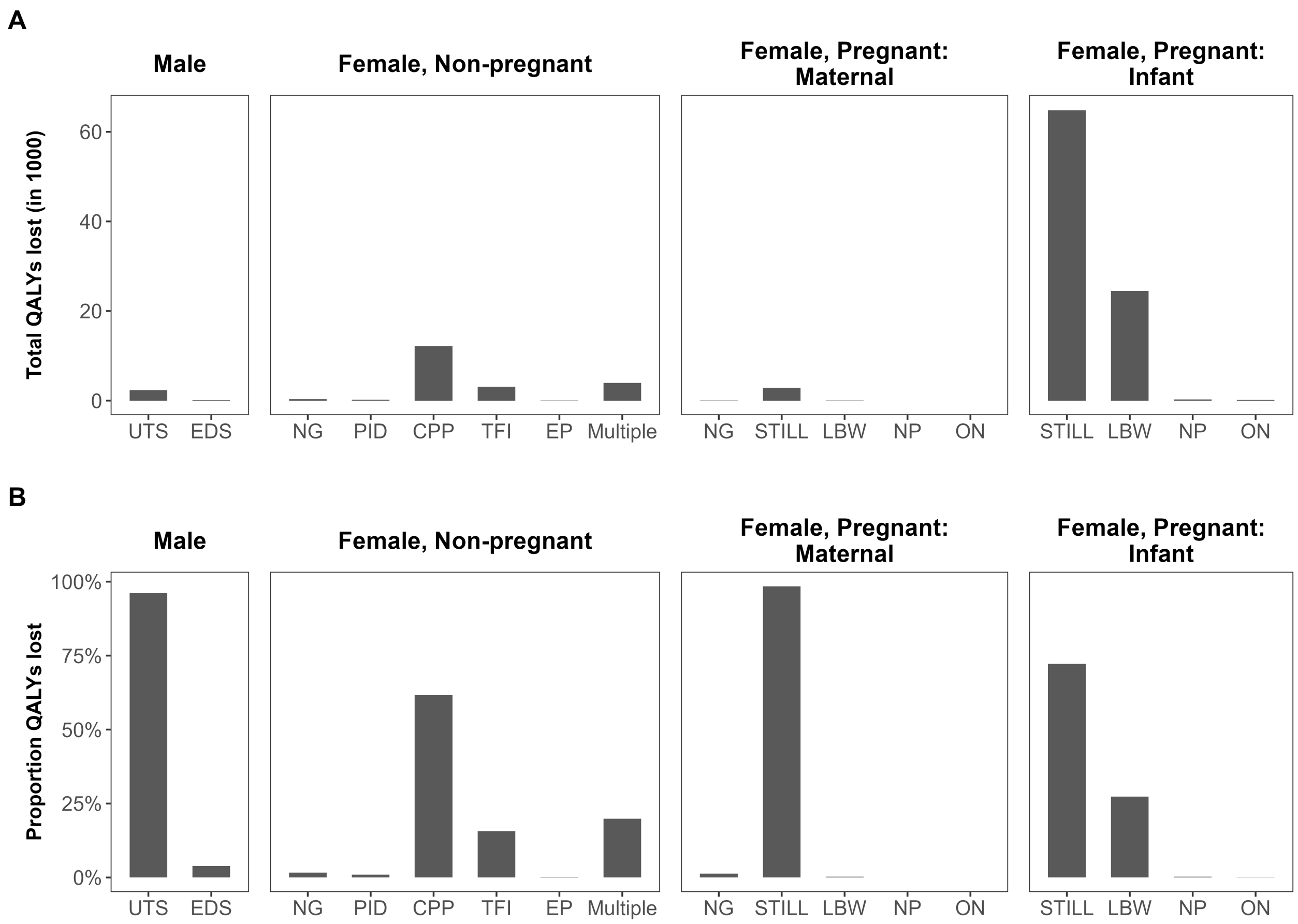

Figure S9: Decomposition of total discounted lifetime QALYs lost

Decomposition of total discounted lifetime QALYs lost per population group as (A) number of QALYs lost and (B) proportional distribution of QALYs lost among each population group. Bars represent median estimates. CPP: chronic pelvic pain, EDS: epididymo-orchitis, EP: ectopic pregnancy, LB: live birth, LBW: low birth weight, NG: *Neisseria gonorrhoeae*, NP: neonatal pneumonia, ON: ophthalmia neonatorum, PID: pelvic inflammatory disease, TFI: tubal factor infertility, UTS: urethritis, Multiple: combined sequelae of CPP, TFI, and EP.

2.3 Intervention outcomes

Table S9: Predicted population-level impact of diagnostic confirmation testing and screening strategies among each priority population with constrained POCT availability, 2025–2030

Variable	CFSW	FSW	Men	Pregnant	AGYW
**Diagnostic confirmation strategy**					
Coverage of POCT among eligible cases* (%)	18.4 (8.55 - 40.2)	66.8 (26.0 - 100)	7.95 (3.91 - 16.2)	33.6 (15.8 - 71.4)	13.1 (5.99 - 28.6)
Percentage of infections averted relative to baseline (%)	2.21 (0.77 - 5.31)	2.07 (0.63 - 5.59)	1.28 (0.46 - 3.03)	1.00 (0.47 - 2.12)	0.93 (0.43 - 1.99)
Percentage of QALYs gained relative to baseline (%)	2.23 (0.78 - 5.28)	2.17 (0.71 - 5.58)	1.29 (0.47 - 3.05)	3.51 (1.75 - 7.22)	0.85 (0.39 - 1.82)
Infections averted per 100 tests	99.8 (45.7 - 165)	94.3 (41.9 - 193)	58.7 (27.1 - 96.3)	44.1 (28.1 - 66.2)	40.6 (26.3 - 64.0)
QALYs gained per 100 tests	10.4 (4.83 - 17.8)	10.1 (5.33 - 20.0)	6.07 (2.90 - 10.1)	16.2 (11.2 - 23.1)	3.80 (2.54 - 5.82)
**Screening strategy**					
Coverage of POCT among eligible cases* (%)	0.98 (0.71 - 1.41)	5.77 (3.90 - 11.1)	0.14 (0.14 - 0.15)	0.82 (0.80 - 0.85)	0.52 (0.47 - 0.60)
Percentage of infections averted relative to baseline (%)	0.58 (0.41 - 0.82)	0.62 (0.20 - 1.52)	0.12 (0.10 - 0.13)	0.08 (0.06 - 0.10)	0.13 (0.11 - 0.16)
Percentage of QALYs gained relative to baseline (%)	0.58 (0.41 - 0.81)	0.65 (0.24 - 1.50)	0.11 (0.10 - 0.13)	0.30 (0.25 - 0.34)	0.12 (0.10 - 0.14)
Infections averted per 100 tests	25.5 (14.6 - 44.2)	26.9 (9.97 - 59.1)	5.04 (3.15 - 8.16)	3.51 (2.04 - 5.97)	5.89 (3.56 - 9.87)
QALYs gained per 100 tests	2.64 (1.44 - 4.70)	2.89 (1.20 - 6.04)	0.52 (0.31 - 0.87)	1.32 (0.80 - 2.42)	0.55 (0.34 - 0.92)

Results presented as medians with uncertainty intervals and rounded to three significant figures. Constrained POCT availability was modelled as 25 000 tests annually. *Cases refer to the number of individuals accessing care for vaginal or urethral discharge (diagnostic scenario) or attending routine healthcare services (screening scenario). Individuals may be counted more than once due to repeat infections or multiple healthcare visits. AGYW: sexually active adolescent girls and young women; CFSW: clients of female sex workers; FSW: female sex workers.

Table S10: Predicted population-level impact of diagnostic confirmation testing and screening strategies among each priority population with unrestricted POCT availability, 2025–2030

Variable	CFSW	FSW	Men	Pregnant	AGYW
**Diagnostic confirmation strategy**					
Average number POCT used annually (×100 000)	1.39 (0.65 - 2.97)	0.38 (0.13 - 0.99)	3.19 (1.59 - 6.46)	0.72 (0.35 - 1.54)	1.96 (0.90 - 4.28)
Percentage of infections averted relative to baseline (%)	11.6 (4.87 - 24.1)	3.07 (0.73 - 10.0)	15.3 (6.81 - 28.9)	3.05 (1.64 - 4.97)	6.93 (3.81 - 10.7)
Percentage of QALYs gained relative to baseline (%)	11.6 (4.87 - 24.1)	3.33 (0.83 - 10.1)	15.4 (6.86 - 28.9)	10.7 (6.43 - 15.8)	6.28 (3.45 - 9.76)
Infections averted per 100 tests	102.0 (46.2 - 171)	93.9 (41.9 - 191)	60.3 (27.4 - 99.8)	43.9 (28.1 - 66.0)	39.7 (25.9 - 62.1)
QALYs gained per 100 tests	10.5 (4.83 - 18.2)	10.1 (5.32 - 20.0)	6.15 (2.92 - 10.5)	16.1 (11.1 - 22.8)	3.73 (2.49 - 5.70)
**Screening strategy**					
Average number POCT used annually (×100 000)	25.4 (18.0 - 34.4)	4.33 (2.25 - 6.38)	175.4 (175.1 - 175.6)	30.4 (29.7 - 31.1)	47.6 (42.8 - 52.1)
Percentage of infections averted relative to baseline (%)	36.3 (27.7 - 42.2)	7.25 (2.16 - 19.5)	45.8 (40.3 - 49.1)	7.59 (6.07 - 9.53)	17.46 (14.6 - 20.5)
Percentage of QALYs gained relative to baseline (%)	36.1 (27.9 - 41.9)	7.65 (2.50 - 19.7)	45.5 (40.1 - 48.5)	28.0 (24.6 - 31.8)	15.9 (13.3 - 18.8)
Infections averted per 100 tests	15.6 (8.48 - 28.6)	19.3 (6.96 - 42.0)	2.84 (1.68 - 4.86)	2.76 (1.60 - 4.73)	4.06 (2.38 - 6.88)
QALYs gained per 100 tests	1.60 (0.85 - 2.95)	2.08 (0.86 - 4.31)	0.29 (0.17 - 0.52)	1.03 (0.62 - 1.90)	0.38 (0.23 - 0.66)

Results presented as medians with uncertainty intervals and rounded to three significant figures. Unrestricted POCT availability was modelled as all eligible cases tested. Cases refer to the number of individuals accessing care for vaginal or urethral discharge (diagnostic scenario) or attending routine healthcare services (screening scenario). Individuals may be counted more than once due to repeat infections or multiple healthcare visits. AGYW: sexually active adolescent girls and young women; CFSW: clients of female sex workers; FSW: female sex workers.

2.4 Sensitivity analyses

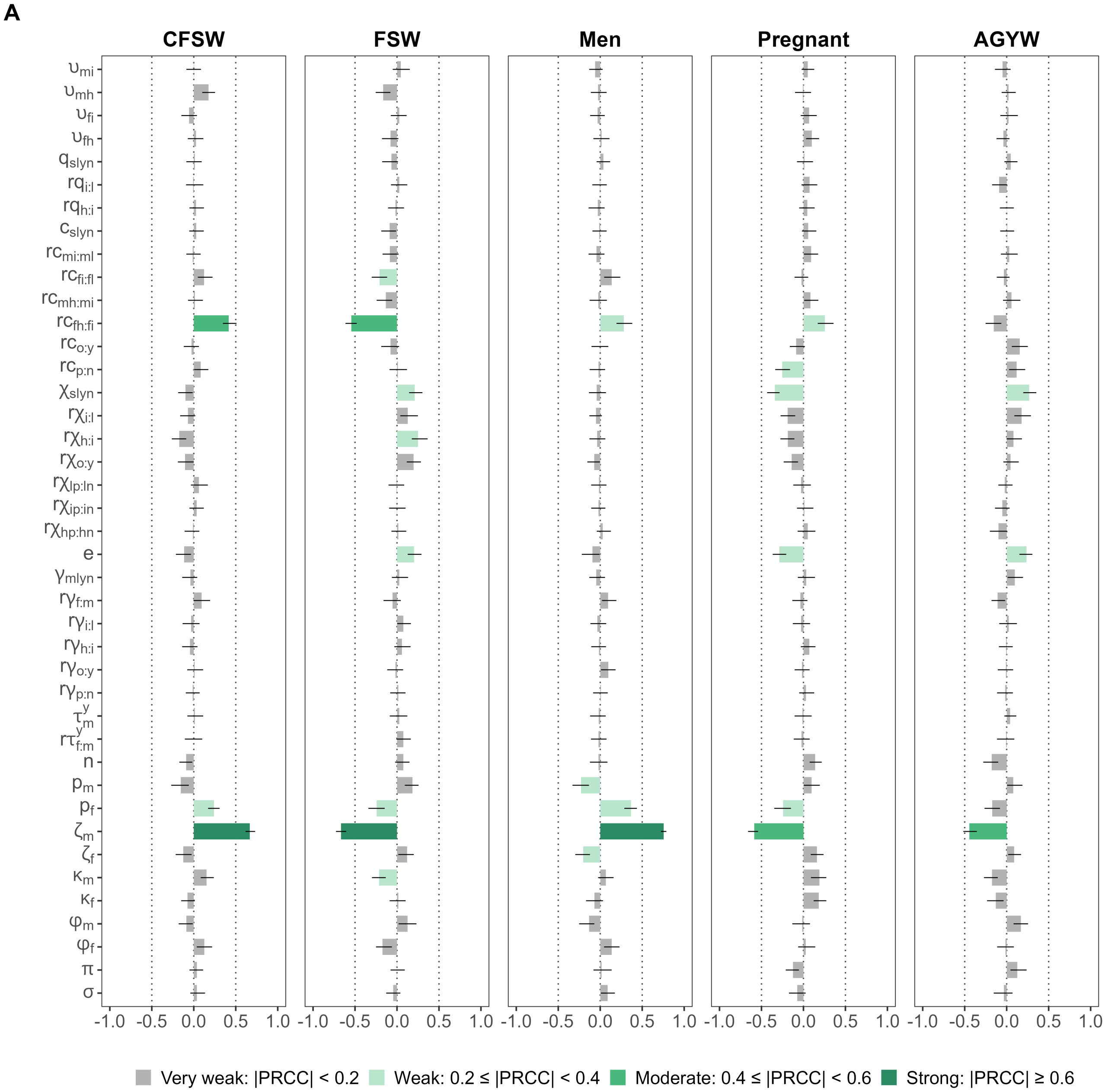

Figure S10: Correlation between parameter values and priority population prioritisation order by test strategy

Partial rank correlation coefficients between posterior parameter values and the prioritisation order of each priority population according to the proportion of incident gonorrhoea infections averted under constrained POCT availability (25 000 annually) between 2025 and 2030 for (A) diagnostic confirmation and (B) screening strategies across each priority population. Bars and lines indicate median and 95% confidence intervals. Parameters are defined in Table S2.

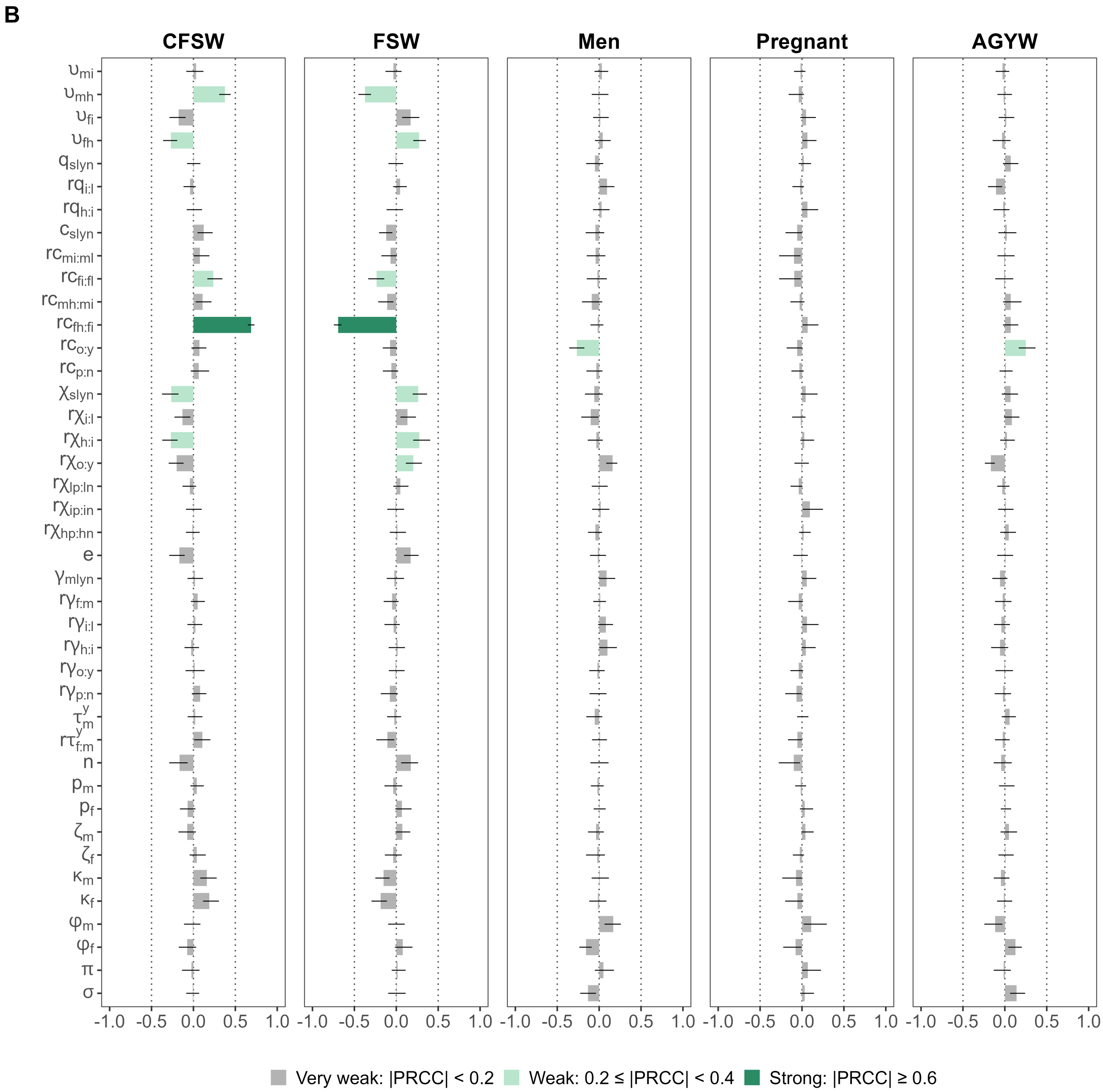

Figure S10: Correlation between parameter values and priority population prioritisation order by test strategy (continued)

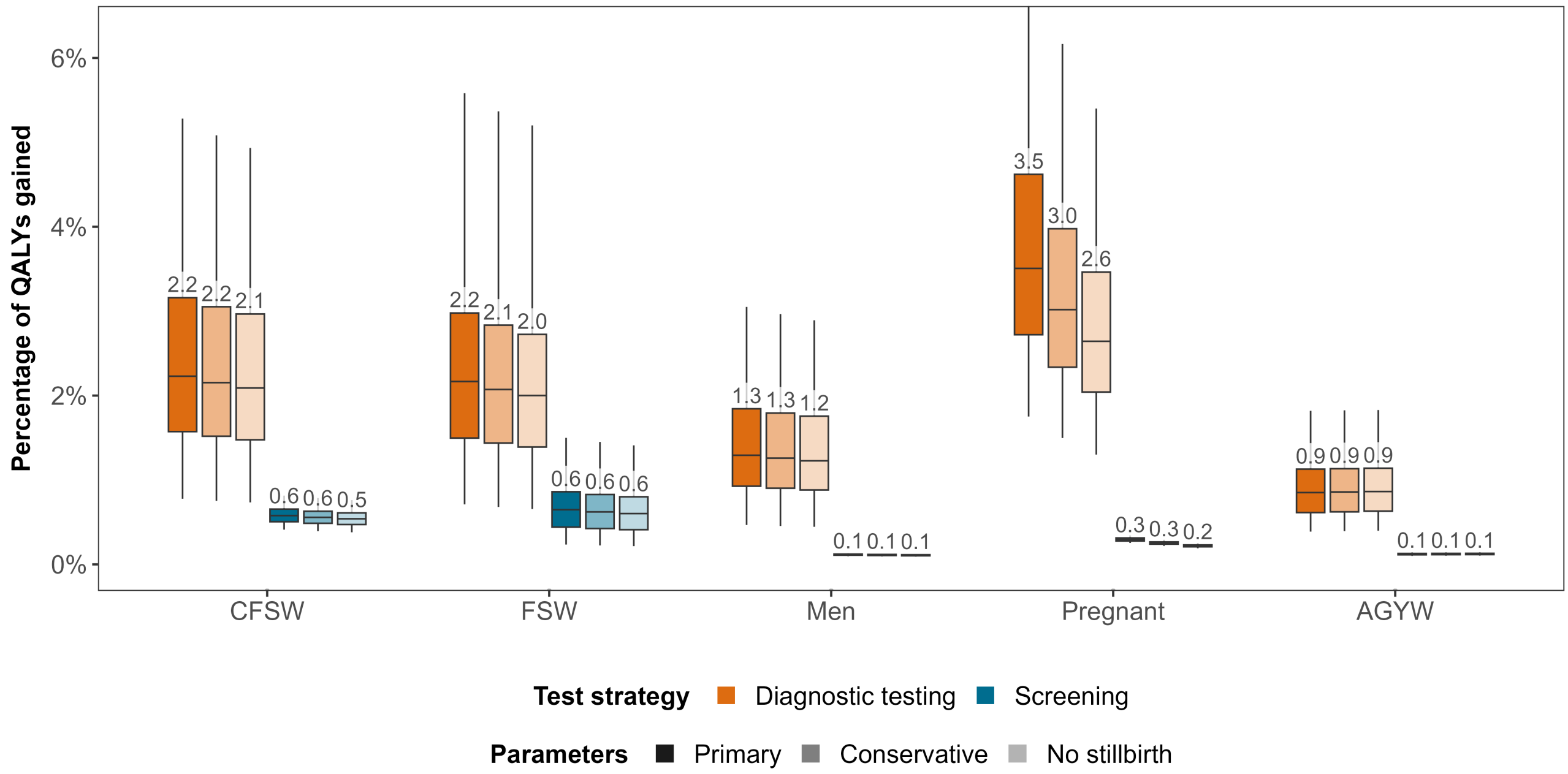

Figure S11: Influence of parameter assumptions for pregnancy-related sequelae on the predicted population-level impact of diagnostic confirmation and screening strategies, 2025–2030

Population-level impact of deploying gonorrhoea POCTs (25 000 annually) among each of five priority populations between 2025 and 2030 through either diagnostic confirmation among individuals accessing care for urethral or vaginal discharge, or screening of attendees at routine healthcare services. Impact was assessed as the percentage of lifetime QALYs gained relative to the baseline scenario of syndromic management only, under three sets of parameter assumptions: (i) Primary, as used in the main analysis, (ii) Conservative, which reduced the lifetime QALY loss duration to 50% of life expectancy for stillbirths and to 10 years for LBW, and (iii) No stillbirth, which excluded stillbirth-associated losses entirely. Parameters describing maternal QALY losses for stillbirth and LBW remained unchanged. Boxplots represent the median (horizontal line), interquartile range (box), and uncertainty interval (whiskers). AGYW: sexually active adolescent girls and young women; CFSW: clients of female sex workers; FSW: female sex workers; POCT: point-of-care test; LBW: low birth weight; QALY: quality-adjusted life year.

Bibliography

1. UNAIDS. The urgency of now: AIDS at a crossroads. Geneva: Joint United Nations Programme on HIV/AIDS; 2024.
2. Garnett GP, Anderson RM. Balancing sexual partnership in an age and activity stratified model of HIV transmission in heterosexual populations. IMA Journal of Mathematics Applied in Medicine & Biology. 1994;11:161-92.
3. UN Population Division. World Population Prospects; 2024. Available from: `https://population.un.org/wpp/`.
4. Kenya National Bureau of Statistics, Kenya Ministry of Health, ORC Macro. Kenya Demographic and Health Survey 2003. Calverton, Maryland: CBS, MOH, and ORC Macro; 2004.
5. Kenya National Bureau of Statistics and ICF Macro. Kenya Demographic and Health Survey 2008-09. Calverton, Maryland: KNBS and ICF Macro; 2010.
6. Kenya National Bureau of Statistics. Kenya Demographic and Health Survey 2014. Rockville, Maryland: KNBS and ICF; 2015.
7. KNBS and ICF. Kenya Demographic and Health Survey 2022. Nairobi, Kenya and Rockville, Maryland, USA: KNBS and ICF; 2023.
8. National AIDS and STI Control Programme. Kenya Population-based HIV Impact Assessment. Nairobi: NASCOP; 2022.
9. Stevens O, Sabin K, Anderson RL, Garcia SA, Willis K, Rao A, et al. Population size, HIV prevalence, and antiretroviral therapy coverage among key populations in sub-Saharan Africa: collation and synthesis of survey data, 2010–23. The Lancet Global Health. 2024 9;12(9):e1400-12.
10. Martin HL Jr, Nyange PM, Richardson BA, Lavreys L, Mandaliya K, Jackson DJ, et al. Hormonal Contraception, Sexually Transmitted Diseases, and Risk of Heterosexual Transmission of Human Immunodeficiency Virus Type 1. The Journal of Infectious Diseases. 1998 10;178(4):1053-9.
11. Fonck K, Kaul R, Kimani J, Keli F, MacDonald KS, Ronald AR, et al. A randomized, placebo-controlled trial of monthly azithromycin prophylaxis to prevent sexually transmitted infections and HIV-1 in Kenyan sex workers: study design and baseline findings. International Journal of STD & AIDS. 2000 12;11(12):804-11.
12. Hawken MP, Melis RDJ, Ngombo DT, Mandaliya K, Ng'ang'a LW, Price J, et al. Part time female sex workers in a suburban community in Kenya: a vulnerable hidden population. Sexually Transmitted Infections. 2002;78(4):271.
13. Cohen CR, Koochesfahani KM, Meier AS, Shen C, Karunakaran K, Ondondo B, et al. Immunoepidemiologic profile of Chlamydia trachomatis infection: Importance of heat-shock protein 60 and interferon-γ. Journal of Infectious Diseases. 2005 8;192(4):591-9.
14. Priddy FH, Wakasiaka S, Hoang TD, Smith DJ, Farah B, Del Rio C, et al. Anal sex, vaginal practices, and HIV incidence in female sex workers in Urban Kenya: Implications for the development of intravaginal HIV prevention methods. AIDS Research and Human Retroviruses. 2011 10;27(10):1067-72.
15. Vandenhoudt HM, Langat L, Menten J, Odongo F, Oswago S, Luttah G, et al. Prevalence of HIV and Other Sexually Transmitted Infections among Female Sex Workers in Kisumu, Western Kenya, 1997 and 2008. PLoS ONE. 2013 1;8(1).
16. Gomih-Alakija A, Ting J, Mugo N, Kwatampora J, Getman D, Chitwa M, et al. Clinical Characteristics Associated with Mycoplasma genitalium among Female Sex Workers in Nairobi, Kenya. Journal of Clinical Microbiology. 2014 10;52(10):3660.
17. Musyoki H, Kellogg TA, Geibel S, Muraguri N, Okal J, Tun W, et al. Prevalence of HIV, Sexually Transmitted Infections, and Risk Behaviours Among Female Sex Workers in Nairobi, Kenya: Results of a Respondent Driven Sampling Study. AIDS and behavior. 2015;19(Suppl 1):S46.

18. Lafort Y, Greener R, Roy A, Greener L, Ombidi W, Lessitala F, et al. HIV prevention and care-seeking behaviour among female sex workers in four cities in India, Kenya, Mozambique and South Africa. Tropical Medicine and International Health. 2016 10;21(10):1293-303.

19. Willcox AC, Richardson BA, Shafi J, Kabare E, Kinuthia J, Jaoko W, et al. Derivation of an HIV Risk Score for African Women Who Engage in Sex Work. AIDS and Behavior. 2021 10;25(10):3292-302.

20. Beksinska A, Jama Z, Kabuti R, Kungu M, Babu H, Nyariki E, et al. Prevalence and correlates of common mental health problems and recent suicidal thoughts and behaviours among female sex workers in Nairobi, Kenya. BMC Psychiatry. 2021 12;21(1):1-17.

21. NASCOP. National behavioral assessment of key populations in Kenya. Polling booth survey report; 2014.

22. NASCOP. National behavioural assessment of key populations in Kenya. Polling booth survey report; 2016.

23. NASCOP. Third national behavioural assessment of key populations in Kenya. Polling booth survey report; 2018.

24. Lockhart A, Psioda M, Ting J, Campbell S, Mugo N, Kwatampora J, et al. Prospective Evaluation of Cervicovaginal Self- and Cervical Physician Collection for the Detection of Chlamydia trachomatis, Neisseria gonorrhoeae, Trichomonas vaginalis, and Mycoplasma genitalium Infections. Sexually transmitted diseases. 2018 7;45(7):488.

25. Warner L, Newman DR, Austin HD, Kamb ML, Douglas JM, Malotte CK, et al. Condom effectiveness for reducing transmission of gonorrhea and chlamydia: the importance of assessing partner infection status. American journal of epidemiology. 2004 2;159(3):242-51.

26. Vickerman P, Watts C, Alary M, Mabey D, Peeling RW. Sensitivity requirements for the point of care diagnosis of Chlamydia trachomatis and Neisseria gonorrhoeae in women. Sexually transmitted infections. 2003 10;79(5):363-8.

27. White RG, Orroth KK, Korenromp EL, Bakker R, Wambura M, Sewankambo NK, et al. Can population differences explain the contrasting results of the Mwanza, Rakai, and Masaka HIV/sexually transmitted disease intervention trials?: A modeling study. Journal of acquired immune deficiency syndromes (1999). 2004 12;37(4):1500-13.

28. Padeniya TN, Hui BB, Wood JG, Seib KL, Regan DG. The potential impact of a vaccine on Neisseria gonorrhoeae prevalence among heterosexuals living in a high prevalence setting. Vaccine. 2023 8;41(38):5553-61.

29. Morison L, Weiss HA, Buvé A, Caraël M, Abega SC, Kaona F, et al. Commercial sex and the spread of HIV in four cities in sub-Saharan Africa. AIDS. 2001 8;15:S61-9.

30. Lafort Y, Greener R, Roy A, Greener L, Ombidi W, Lessitala F, et al. Where do female sex workers seek HIV and reproductive health care and what motivates these choices? A survey in 4 cities in India, Kenya, Mozambique and South Africa. PLoS ONE. 2016 8;11(8).

31. Wallin J. Gonorrhoea in 1972. A 1-year study of patients attending the VD Unit in Uppsala. British Journal of Venereal Diseases. 1974;51:41.

32. Harrison WO, Hooper RR, Wiesner PJ, Campbell AF, Karney WW, Reynolds GH, et al. A trial of minocycline given after exposure to prevent gonorrhea. The New England journal of medicine. 1979 5;300(19):1074-8.

33. Platt R, Rice PA, McCormack WM. Risk of acquiring gonorrhea and prevalence of abnormal adnexal findings among women recently exposed to gonorrhea. JAMA. 1983 12;250(23):3205-9.

34. Sherrard J, Barlow D. Men with repeated episodes of gonorrhoea 1990-1992. International Journal of STD & AIDS. 1996 7;7(4):281-2.

35. Garnett P, Mertz KJ, Finelli L, Levine WC, St Louis ME. The transmission dynamics of gonorrhoea: modelling the reported behaviour of infected patients from Newark, New Jersey. The Royal Society. 1999;354:787-97.

36. Korenromp EL, Sudaryo MK, De Vlas SJ, Gray RH, Sewankambo NK, Serwadda D, et al. What proportion of episodes of gonorrhoea and chlamydia becomes symptomatic? International Journal of STD and AIDS. 2002;13(2):91-101.

37. Meyer-Weitz A, Reddy P, Van Den Borne HW, Kok G, Pietersen J. Health care seeking behaviour of patients with sexually transmitted diseases: determinants of delay behaviour. Patient Education and Counseling. 2000 10;41(3):263-74.

38. Fonck K, Mwai C, Ndinya-Achola J, Bwayo J, Temmerman M. Health-seeking and sexual behaviors among primary healthcare patients in Nairobi, Kenya. Sexually transmitted diseases. 2002;29(2):106-11.

39. Voeten H, O'Hara HB, Kusimba J, Otido JM, Ndinya-Achola JO, Bwayo JJ, et al. Gender differences in health care-seeking behavior for sexually transmitted diseases: a population-based study in Nairobi, Kenya. Sexually Transmitted Diseases. 2004 5;31(5):265-72.

40. Govender I, Eche M. Health-seeking behaviour of people with sexually transmitted infections in the community of Nkomazi East, Mpumalanga. Southern African Journal of Epidemiology and Infection. 2012 1;27(4):195-8.

41. Nyalela M, Dlungwane T, Taylor M, Nkwanyana N. Health seeking and sexual behaviour of men presenting with sexually transmitted infections in two primary health care clinics in Durban. Southern African Journal of Infectious Diseases. 2018 10:1-6.

42. Wambiya EOA, Otieno PO, Mutua MK, Donfouet HPP, Mohamed SF. Patterns and predictors of private and public health care utilization among residents of an informal settlement in Nairobi, Kenya: a cross-sectional study. BMC Public Health. 2021 12;21(1):850.

43. Leslie HH, Malata A, Ndiaye Y, Kruk ME. Effective coverage of primary care services in eight high-mortality countries. BMJ Global Health. 2017 9;2(3).

44. Kenya institute for public policy research and analysis. An assessment of healthcare delivery in Kenya under the devolved system. Special paper no. 19; 2018.

45. Kenya Ministry of Health. Kenya health facility census report. Nairobi: Kenya Ministry of Health; 2023.

46. Otieno PO, Kiroro F, Runyenje C, Kamau P. Unmet need for primary healthcare and associated individual and household-level factors in Kenya: results from a national survey. BMJ Open. 2021;11:41032.

47. Parmley L, Rao A, Kose Z, Lambert A, Max R, Phaswana-Mafuya N, et al. Antenatal care presentation and engagement in the context of sex work: Exploring barriers to care for sex worker mothers in South Africa. Reproductive Health. 2019 5;16(1):1-12.

48. Moore BE, Govaerts L, Kapadia F. Maternal health and maternal health service utilization among female sex workers: A scoping review. Women's Health. 2023 1;19.

49. World Health Organization. Guidelines for the management of symptomatic sexually transmitted infections. Web Annex A. Syndromic management or point of care tests for urethral discharge: systematic review and mathematical modelling. Geneva: World Health Organization; 2021.

50. Zemouri C, Wi TE, Kiarie J, Seuc A, Mogasale V, Latif A, et al. The performance of the vaginal discharge syndromic management in treating vaginal and cervical infection: A systematic review and meta-analysis. PLoS ONE. 2016 10;11(10):e0163365.

51. Wi TE, Ndowa FJ, Ferreyra C, Kelly-Cirino C, Taylor MM, Toskin I, et al. Diagnosing sexually transmitted infections in resource-constrained settings: challenges and ways forward. Journal of the International AIDS Society. 2019 8;22(Suppl 6):e25343.

52. Ferreyra C, Osborn J, Moussy F, Alirol E, Lahra M, Whiley D, et al. Developing target product profiles for Neisseria gonorrhoeae diagnostics in the context of antimicrobial resistance: An expert consensus. PLOS ONE. 2020 9;15(9):e0237424.

53. Holmes KK, Johnson DW, Trostle HJ. An estimate of the risk of men acquiring gonorrhea by sexual contact with infected females. American Journal of Epidemiology. 1970 2;91(2):170-4.

54. Hooper RR, Reynolds GH, Jones OG, Zaidl A, Wtesner PJ, Latimer KP, et al. Cohort study of venereal disease. The risk of gonorrhea transmission from infected women to men. American Journal of Epidemiology. 1978;108(2).

55. Hethcote HW, Yorke JA. Gonorrhea transmission dynamics and control; 1984. Available from: http://yorke.umd.edu/papers/Hethcote-Yorke-Gonorrhea-Transmission-and-Control-1984.pdf.

56. Johnson LF, Geffen N. A comparison of two mathematical modeling frameworks for evaluating sexually transmitted infection epidemiology. Sexually Transmitted Diseases. 2016;43(3):139-46.

57. Orroth KK, Freeman EE, Bakker R, Buvé A, Glynn JR, Boily MC, et al. Understanding the differences between contrasting HIV epidemics in east and west Africa: results from a simulation model of the Four Cities Study. Sexually transmitted infections. 2007 8;83(Suppl. 1).

58. Wiesner PJ, Thompson SE. Gonococcal diseases. Year Book Medical Publishers; 1980.

59. Farley TA, Cohen DA, Elkins W. Asymptomatic sexually transmitted diseases: the case for screening. Preventive Medicine. 2003 4;36(4):502-9.

60. Fortas C, Delarocque-Astagneau E, Randremanana RV, Crucitti T, Huynh BT. Asymptomatic infections with Chlamydia trachomatis, Neisseria gonorrhoeae, and Trichomonas vaginalis among women in low- and middle-income countries: A systematic review and meta-analysis. PLOS global public health. 2024 5;4(5).

61. Brunham RC, Garnett GP, Swinton J, Anderson RM. Gonococcal infection and human fertility in sub-Saharan Africa. Proceedings of the Royal Society B: Biological Sciences. 1991;246(1316):173-7.

62. Hui BB, Wilson DP, Ward JS, Guy RJ, Kaldor JM, Law MG, et al. The potential impact of new generation molecular point-of-care tests on gonorrhoea and chlamydia in a setting of high endemic prevalence. Sexual Health. 2013;10(4):348-56.

63. Hazel A, Marino S, Simon C. An anthropologically based model of the impact of asymptomatic cases on the spread of Neisseria gonorrhoeae. Journal of the Royal Society, Interface. 2015 5;12(106).

64. World Health Organization. Guidelines for the management of symptomatic sexually transmitted infections. Geneva: World Health Organization; 2021.

65. Michalow J, Walters MK, Olanrewaju E, Wybrant M, Davies B, Kufa T, et al. Aetiology of vaginal discharge, urethral discharge, and genital ulcer in sub-Saharan Africa: A systematic review and meta-regression. PLOS Medicine. 2024 5;21(5):e1004385.

66. Michalow J, Hall L, Rowley J, Anderson RL, Hayre Q, Chico RM, et al. Prevalence of chlamydia, gonorrhoea, and trichomoniasis among male and female general populations in sub-Saharan Africa from 2000 to 2024: a systematic review and meta-regression analysis. eClinicalMedicine. 2025 5;83:103210.

67. Hawken MP, Melis RDJ, Ngombo DT, Mandaliya KN, Ng'ang'a LW, Price J, et al. Opportunity for Prevention of HIV and Sexually Transmitted Infections in Kenyan Youth: Results of a Population-Based Survey. JAIDS Journal of Acquired Immune Deficiency Syndromes. 2002 12;31(5):529-35.

68. Otieno FO, Ndivo R, Oswago S, Pals S, Chen R, Thomas T, et al. Correlates of prevalent sexually transmitted infections among participants screened for an HIV incidence cohort study in Kisumu, Kenya. International Journal of STD and AIDS. 2015 3;26(4):225-37.

69. Jespers V, Crucitti T, Menten J, Verhelst R, Mwaura M, Mandaliya K, et al. Prevalence and Correlates of Bacterial Vaginosis in Different Sub-Populations of Women in Sub-Saharan Africa: A Cross-Sectional Study. PLoS ONE. 2014 10;9(10).

70. Kinuthia J, Drake AL, Matemo D, Richardson BA, Zeh C, Osborn L, et al. HIV Acquisition During Pregnancy and Postpartum is Associated with Genital Infections and Partnership Characteristics: A Cohort Study. AIDS. 2015 9;29(15).

71. Kerubo E, Laserson KF, Otecko N, Odhiambo C, Mason L, Nyothach E, et al. Prevalence of reproductive tract infections and the predictive value of girls' symptom-based reporting: findings from a cross-sectional survey in rural western Kenya. Sexually Transmitted Infections. 2016 6;92(4):251-6.

72. Maina AN, Kimani J, Anzala O. Prevalence and risk factors of three curable sexually transmitted infections among women in Nairobi, Kenya. BMC Research Notes. 2016;9(1).

73. Oliver VO, Otieno G, Gvetadze R, Desai MA, Makanga M, Akelo V, et al. High prevalence of sexually transmitted infections among women screened for a contraceptive intravaginal ring study, Kisumu, Kenya, 2014. International Journal of STD and AIDS. 2018 12;29(14):1390-9.

74. Masha SC, Wahome E, Vaneechoutte M, Cools P, Crucitti T, Sanders EJ. High prevalence of curable sexually transmitted infections among pregnant women in a rural county hospital in Kilifi, Kenya. PLoS ONE. 2017 3;12(3).

75. Yuh T, Micheni M, Selke S, Oluoch L, Kiptinness C, Magaret A, et al. Sexually Transmitted Infections Among Kenyan Adolescent Girls and Young Women With Limited Sexual Experience. Frontiers in Public Health. 2020 7;8.

76. Deese J, Philip N, Lind M, Ahmed K, Batting J, Beksinska M, et al. Sexually transmitted infections among women randomised to depot medroxyprogesterone acetate, a copper intrauterine device or a levonorgestrel implant. Sexually Transmitted Infections. 2021 6;97(4):249-55.

77. Mgodi NM, Takuva S, Edupuganti S, Karuna S, Andrew P, Lazarus E, et al. A Phase 2b Study to Evaluate the Safety and Efficacy of VRC01 Broadly Neutralizing Monoclonal Antibody in Reducing Acquisition of HIV-1 Infection in Women in Sub-Saharan Africa: Baseline Findings. JAIDS Journal of Acquired Immune Deficiency Syndromes. 2021 5;87(1):680-7.

78. Madanitsa M, Barsosio HC, Minja DTR, Mtove G, Kavishe RA, Dodd J, et al. Effect of monthly intermittent preventive treatment with dihydroartemisinin–piperaquine with and without azithromycin versus monthly sulfadoxine–pyrimethamine on adverse pregnancy outcomes in Africa: a double-blind randomised, partly placebo-controlled trial. The Lancet. 2023 3;401(10381):1020-36.

79. Mehta SD, Zulaika G, Agingu W, Nyothach E, Bhaumik R, Green SJ, et al. Analysis of bacterial vaginosis, the vaginal microbiome, and sexually transmitted infections following the provision of menstrual cups in Kenyan schools: Results of a nested study within a cluster randomized controlled trial. PLoS Medicine. 2023 7;20(7 July).

80. Lokken EM, Jisuvei C, Oyaro B, Shafi J, Nyaigero M, Kinuthia J, et al. Nugent Score, Amsel's Criteria, and a Point-of-Care Rapid Test for Diagnosis of Bacterial Vaginosis: Performance in a Cohort of Kenyan Women. Sexually Transmitted Diseases. 2022 1;49(1):E22-5.

81. Celum CL, Bukusi EA, Bekker LG, Delany-Moretlwe S, Kidoguchi L, Omollo V, et al. PrEP use and HIV seroconversion rates in adolescent girls and young women from Kenya and South Africa: the POWER demonstration project. Journal of the International AIDS Society. 2022 7;25(7):25962.

82. Heffron R, Casmir E, Aswani L, Ngure K, Kwach B, Ogello V, et al. HIV risk and pre-exposure prophylaxis interest among women seeking post-abortion care in Kenya: a cross-sectional study. Journal of the International AIDS Society. 2021 5;24(5):25703.

83. Oware K, Adiema L, Rono B, Violette LR, McClelland RS, Donnell D, et al. Characteristics of Kenyan women using HIV PrEP enrolled in a randomized trial on doxycycline postexposure prophylaxis for sexually transmitted infection prevention. BMC women's health. 2023 12;23(1).

84. Mbuvi CM, Musila BN, Nyamache AK. Urogenital Infections Among Women Attending Mwingi Hospital, Kitui County, Kenya: Safeguarding Antibiotics Through Microbiological Diagnosis. East Afr Health Res J. 2024;8(1):99-105.

85. Bailey RC, Moses S, Parker CB, Agot K, Maclean I, Krieger JN, et al. Male circumcision for HIV prevention in young men in Kisumu, Kenya: a randomised controlled trial. Lancet. 2007 2;369(9562):643-56.

86. Cohen CR, Nosek M, Meier A, Astete SG, Iverson-Cabral S, Mugo NR, et al. Mycoplasma genitalium infection and persistence in a cohort of female sex workers in Nairobi, Kenya. Sexually Transmitted Diseases. 2007 5;34(5):274-9.

87. Kwena ZA, Bukusi EA, Ng'ayo MO, Buffardi AL, Nguti R, Richardson B, et al. Prevalence and risk factors for sexually transmitted infections in a high-risk occupational group: The case of fishermen along Lake Victoria in Kisumu, Kenya. International Journal of STD and AIDS. 2010 10;21(10):708-13.

88. Chersich MF, Luchters SMF, Malonza IM, Mwarogo P, King'ola N, Temmerman M. Heavy episodic drinking among Kenyan female sex workers is associated with unsafe sex, sexual violence and sexually transmitted infections. International Journal of STD & AIDS. 2007 11;18(11):764-9.

89. Izulla P, McKinnon LR, Munyao J, Karanja S, Koima W, Parmeres J, et al. HIV postexposure prophylaxis in an urban population of female sex workers in Nairobi, Kenya. Journal of Acquired Immune Deficiency Syndromes. 2013 2;62(2):220-5.

90. Grabert BK, Islam JY, Kabare E, Vielot NA, Waweru W, Mandaliya K, et al. Testing for Sexually Transmitted Infection Using Wet and Dry Self-Collected Brush Samples Among Women in Mombasa, Kenya. Sexually Transmitted Diseases. 2022 9;49(9):E100-3.
91. Li Y, You S, Lee K, Yaesoubi R, Hsu K, Gift TL, et al. The Estimated Lifetime Quality-Adjusted Life-Years Lost Due to Chlamydia, Gonorrhea, and Trichomoniasis in the United States in 2018. The Journal of infectious diseases. 2023 4;227(8):1007-18.
92. Li Y, Rönn MM, Tuite AR, Chesson HW, Gift TL, Trikalinos TA, et al. Estimated costs and quality-adjusted life-years lost due to N. gonorrhoeae infections acquired in 2015 in the United States: A modelling study of overall burden and disparities by age, race/ethnicity, and other factors. The Lancet Regional Health - Americas. 2022 12;16:100364.
93. Institute for Health Metrics and Evaluation. Global Burden of Disease Study 2019 (GBD 2019) Disability Weights; 2020.
94. World Health Organization. The global burden of disease: 2004 update. Geneva: World Health Organization; 2008.
95. Institute of Medicine (US) Committee to Study Priorities for Vaccine Development. Vaccines for the 21st Century: A Tool for Decisionmaking. Stratton KR, Durch JS, Lawrence RS, editors. Washington: National Academies Press (US); 2000.
96. Aledort JE, Hook EW, Weinstein MC, Goldie SJ. The cost effectiveness of gonorrhea screening in urban emergency departments. Sexually transmitted diseases. 2005 7;32(7):425-36.
97. Jamison DT, Shahid-Salles SA, Jamison J, Lawn JE, Zupan J. Incorporating Deaths Near the Time of Birth into Estimates of the Global Burden of Disease. Lopez A, Mathers C, Ezzati M, Jamison D, Murray CJL, editors. New York: Oxford University Press; 2006.
98. Camacho EM, Gold KJ, Murphy M, Storey C, Heazell AEP. Measuring EQ-5D-5L utility values in parents who have experienced perinatal death. The European Journal of Health Economics. 2024 11;25(8).
99. Lee K, You S, Li Y, Chesson H, Gift TL, Berruti AA, et al. Estimation of the Lifetime Quality-Adjusted Life Years (QALYs) Lost Due to Syphilis Acquired in the United States in 2018. Clinical Infectious Diseases. 2023 2;76(3):E810-9.
100. Ong JJ, Chen M, Hocking J, Fairley CK, Carter R, Bulfone L, et al. Chlamydia screening for pregnant women aged 16-25 years attending an antenatal service: a cost-effectiveness study. BJOG. 2016 6;123(7):1194-202.
101. Trikalinos T. Technical Appendix: Probability of pelvic inflammatory disease in Chlamydia trachomatis infection; 2018.
102. Blencowe H, Cousens S, Jassir FB, Say L, Chou D, Mathers C, et al. National, regional, and worldwide estimates of stillbirth rates in 2015, with trends from 2000: A systematic analysis. The Lancet Global Health. 2016 2;4(2):e98-e108.
103. Blencowe H, Krasevec J, de Onis M, Black RE, An X, Stevens GA, et al. National, regional, and worldwide estimates of low birthweight in 2015, with trends from 2000: a systematic analysis. The Lancet Global Health. 2019 7;7(7):e849-60.
104. Vallely LM, Egli-Gany D, Wand H, Pomat WS, Homer CSE, Guy R, et al. Adverse pregnancy and neonatal outcomes associated with Neisseria gonorrhoeae: systematic review and meta-analysis. Sex Transm Infect. 2021;97:104-11.
105. Nyemba DC, Joseph-Davey DL, Delany-Moretlwe S, Myer L, Johnson LF. The effect of STI screening during pregnancy on vertical transmission of HIV and adverse pregnancy outcomes in South Africa: a modelling study. Journal of the International AIDS Society. 2025 2;28(2):e26410.
106. Miao P, Terris-Prestholt F, Fairley CK, Tucker JD, Wiseman V, Mayaud P, et al. Ignored and undervalued in public health: a systematic review of health state utility values associated with syphilis infection. Health and Quality of Life Outcomes. 2024 12;22(1):1-21.
107. Institute of Medicine. Preterm birth: Causes, consequences, and prevention. Behrman RE, Butler AS, editors. Washington, DC: National Academies Press; 2007.

108. van der Pal S, Steinhof M, Grevinga M, Wolke D, Verrips GE. Quality of life of adults born very preterm or very low birth weight: A systematic review. Acta paediatrica. 2020 10;109(10):1974-88.

109. Murray CJL, Ezzati M, Flaxman AD, Lim S, Lozano R, Michaud C, et al. GBD 2010: design, definitions, and metrics. The Lancet. 2012 12;380(9859):2063-6.

110. World Health Organization Department of Data and Analytics. WHO methods and data sources for global burden of disease estimates 2000-2019. Geneva: World Health Organization; 2020.
